# Synthesis, Antiplasmodial, and Antileukemia Activity of Dihydroartemisinin–HDAC Inhibitor Hybrids as Multitarget Drugs

**DOI:** 10.3390/ph15030333

**Published:** 2022-03-09

**Authors:** Lukas von Bredow, Thomas Martin Schäfer, Julian Hogenkamp, Maik Tretbar, Daniel Stopper, Fabian B. Kraft, Julian Schliehe-Diecks, Andrea Schöler, Arndt Borkhardt, Sanil Bhatia, Jana Held, Finn K. Hansen

**Affiliations:** 1 Medical Faculty, Institute for Drug Discovery, Leipzig University, 04103 Leipzig, Germany; jv51hyza@studserv.uni-leipzig.de (L.v.B.); maik.tretbar@uni-leipzig.de (M.T.); andrea.schoeler@uni-leipzig.de (A.S.); 2 Institut für Tropenmedizin, Eberhard Karls Universität Tübingen, 72074 Tübingen, Germany; thomas-martin.schaefer@student.uni-tuebingen.de (T.M.S.); jana.held@uni-tuebingen.de (J.H.); 3 Department of Pediatric Oncology, Hematology and Clinical Immunology, Medical Faculty, Heinrich-Heine University Düsseldorf, 40225 Düsseldorf, Germany; julian.hogenkamp@med.uni-duesseldorf.de (J.H.); julian.schliehe-diecks@med.uni-duesseldorf.de (J.S.-D.); arndt.borkhardt@med.uni-duesseldorf.de (A.B.); sanil.bhatia@med.uni-duesseldorf.de (S.B.); 4 Department of Pharmaceutical and Cell Biological Chemistry, Pharmaceutical Institute, University of Bonn, 53121 Bonn, Germany; dstopper@uni-bonn.de (D.S.); fkraft@uni-bonn.de (F.B.K.); 5 German Center for Infection Research (DZIF), Partner Site Tübingen, 72074 Tübingen, Germany

**Keywords:** histone deacetylase, artemisinin, multitarget drugs

## Abstract

Artemisinin-based combination therapies (ACTs) are the gold standard for the treatment of malaria, but the efficacy is threatened by the development of parasite resistance. Histone deacetylase inhibitors (HDACis) are an emerging new class of potential antiplasmodial drugs. In this work, we present the design, synthesis, and biological evaluation of a mini library of dihydroartemisinin–HDACi hybrid molecules. The screening of the hybrid molecules for their activity against selected human HDAC isoforms, asexual blood stage *P. falciparum* parasites, and a panel of leukemia cell lines delivered important structure–activity relationships. All synthesized compounds demonstrated potent activity against the 3D7 and Dd2 line of *P. falciparum* with IC_50_ values in the single-digit nanomolar range. Furthermore, the hybrid (α)-**7c** displayed improved activity against artemisinin-resistant parasites compared to dihydroartemisinin. The screening of the compounds against five cell lines from different leukemia entities revealed that all hydroxamate-based hybrids (**7a–e**) and the *ortho*-aminoanilide **8** exceeded the antiproliferative activity of dihydroartemisinin in four out of five cell lines. Taken together, this series of hybrid molecules represents an excellent starting point toward the development of antimalarial and antileukemia drug leads.

## 1. Introduction

Malaria is a tropical disease caused by different species of the *Plasmodium* genus. In 2019, it accounted for 409,000 deaths among a total of 229 million cases, most of which were caused by *P. falciparum*, the most dangerous parasite [1]. Artemisinin-based combination therapies (ACTs) are the current gold standard of malaria treatments [2], but the rapid development of drug resistance remains an issue to be tackled by new therapeutic approaches [3]. To avoid cross-resistance, new antimalarials should engage in novel modes of action that are capable of targeting the parasite at different life cycle stages [4]. A well-known strategy to identify new therapeutic agents is the “piggyback” approach focusing on drug targets that have been validated for other diseases. Using this method, multiple research groups have identified histone deacetylase inhibitors (HDACis) as promising new antiplasmodial compounds [5,6]. Impacting the acetylation levels of histones and other substrates, HDACs control the transcriptional regulation of genes in eukaryotes and have thus become valuable targets in cancer therapy [7]. With five HDAC isoforms being prevalent in *P. falciparum* [8,9,10], HDACis have also shown potential as antimalarial drugs in the preclinical stage [5,11,12,13,14,15,16].

Using two or more separate drugs, combination therapies are effective for the treatment of complex diseases, such as malaria, cancer, HIV, or tuberculosis [17]. One benefit of such drug cocktails is the possibility of suppressing resistance-driving mechanisms, but due to different pharmacokinetic profiles and possible unwanted drug-drug interactions, the scope of combination therapies may turn out to be limited [18]. A promising alternative is the concept of polypharmacology, which aims to serve two individual modes of action using only one drug [19,20]. Typically designed by merging two pharmacophores into a single molecule, such compounds offer the advantage of a more predictable metabolic behavior and simultaneous occupation of each target [20]. Suitable synergistic effects between the respective targets can moreover be addressed to circumvent drug resistance [17,21]. With this complication being particularly relevant in malaria therapy, polypharmacological HDACis may emerge as a new class of antiplasmodial drugs. 

In the past years, numerous artemisinin-derived hybrids have been synthesized and investigated for their biological activity [22,23]. However, only one report on artemisinin–HDACi hybrids have been published so far, and to the best of our knowledge, none of the compounds was tested for its antiplasmodial properties [24]. In this study, we present the synthesis and biological evaluation of newly designed dihydroartemisinin-based HDACi hybrid drugs. All compounds were screened for their inhibition of human HDAC1 and HDAC6. Moreover, cellular HDAC inhibitory activity was tested with a representative dihydroartemisinin-based HDACi. In vitro assays against the *P. falciparum* strains 3D7 and Dd2 demonstrated promising antiplasmodial potential with IC_50_ values ranging in the single-digit nanomolar concentration range. Selected compounds were further tested for their activity against artemisinin-resistant parasites. Furthermore, we investigated the antileukemia activity of this series of compounds using five cell lines from different leukemia entities. The most promising compounds were further tested for their ability to induce apoptosis in leukemia cells.

## 2. Results and Discussion

### 2.1. Design and Synthesis of Dihydroartemisinin–HDACi Hybrids

**Figure 1:**
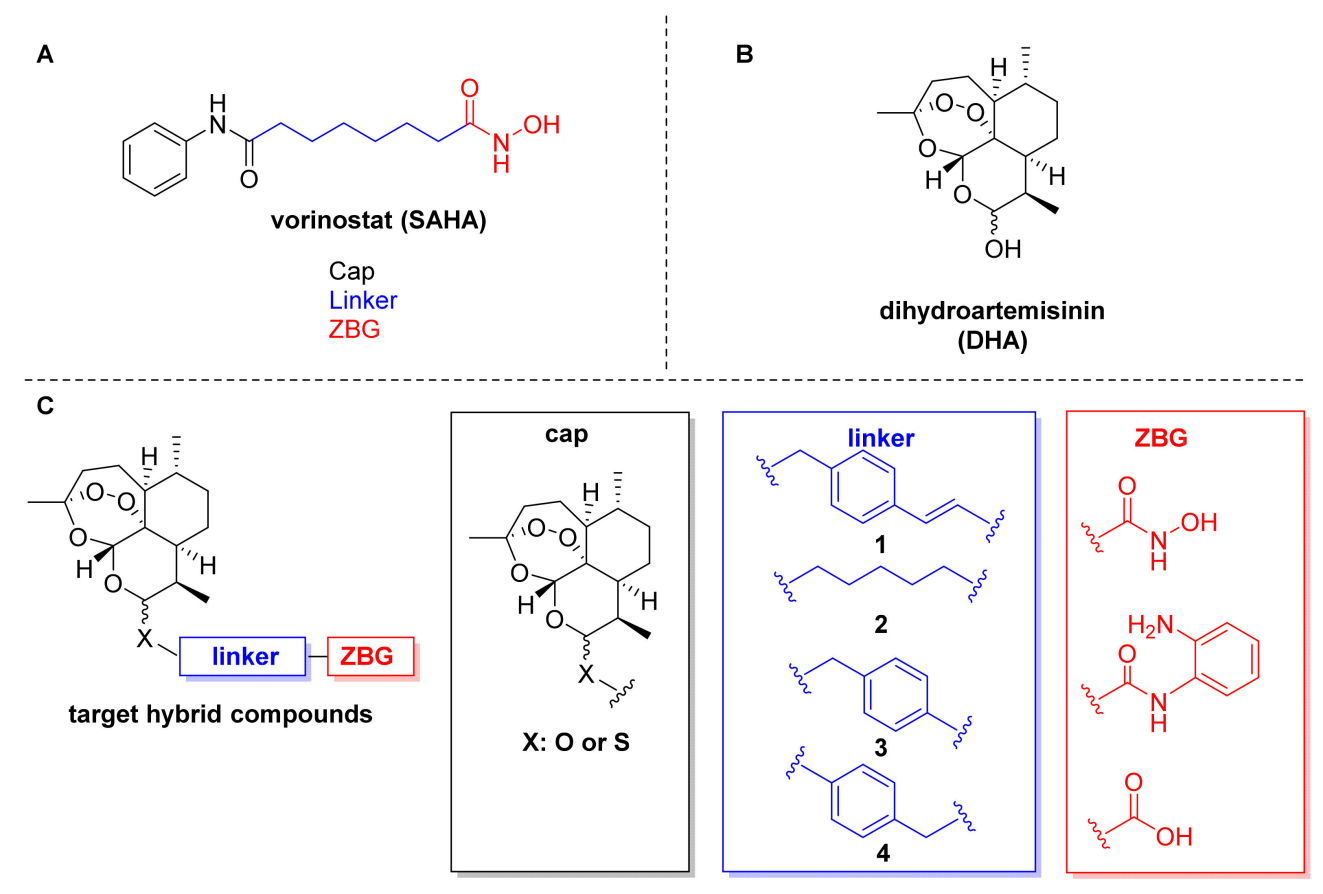
(**A**) HDACi pharmacophore model illustrated on the FDA-approved drug vorinostat (SAHA). (**B**) Chemical structure of dihydroartemisinin (DHA). (**C**) Compound design based on DHA as cap connected by C-10 ether or thioether groups to four different linkers and three zinc-binding groups.

The typical structure of HDACis can be described by a simple cap−linker−zinc-binding group pharmacophore model (Figure 1A). The zinc-binding group (ZBG) chelates the Zn^2+^ ion in the active site of the enzymes, whereas the cap group engages the entrance area of the catalytic site. Both parts are connected by a suitable linker, which interacts with hydrophobic amino acids inside of the catalytic tunnel. This pharmacophore model tolerates a variety of different cap groups including macrocycles [25]. Consequently, we selected dihydroartemisinin (DHA, Figure 1B) as a suitable cap group for our hybrid compounds. Our compound design is summarized in Figure 1C. To incorporate some diversity at the connecting unit between the cap and linker, we designed C-10 ether (X = O) and C-10 thioether (X = S) derivatives. Overall, four different linker groups were chosen including vinylbenzyl (**1**), alkyl (**2**), and benzyl (**3**, **4**). The ZBG is crucial for the biological activity of HDACis, and the vast majority of antiplasmodial HDACis utilize a hydroxamic acid such as ZBG [26]. Thus, we decided to focus on hydroxamates. However, to explore alternative ZBG’s, we also included one *o*-aminoanilide and one carboxylic acid into the compound design.

**Scheme 1:**
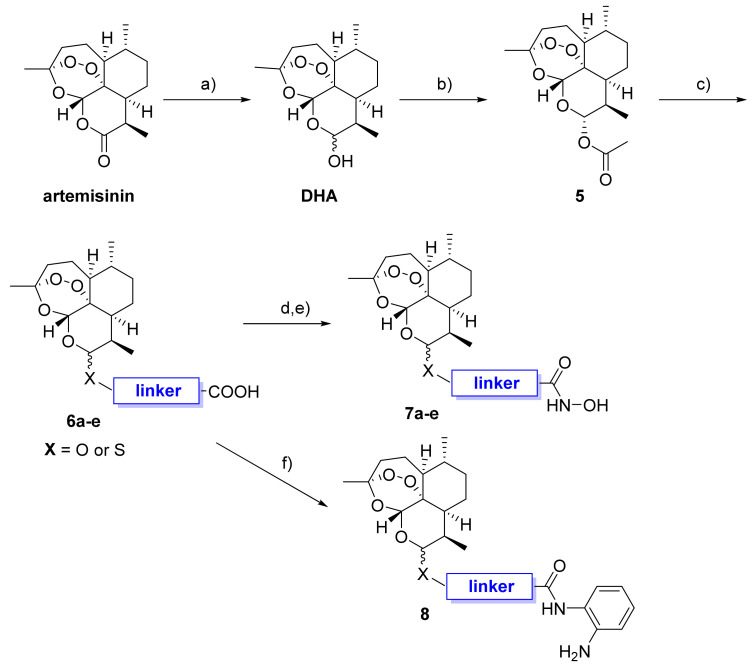
Synthesis of DHA–HDACi hybrids: (**a**) NaBH_4_ (2.5 equiv.), MeOH, 0 °C → r.t., 0.5 h, 98%. (**b**) Ac_2_O, pyridine, r.t., 20 h, 92% (**c**) HX-linker-COOH (1.2 equiv.), BF_3_ OEt_2_ (1.05 equiv.), DCM, −15 °C, 0.5 h, 55–75%. (**d**) NH_2_-O-THP (1.0 equiv.), EDC.HCl (1.0 equiv.), DMAP (0.5 equiv.), DCM, r.t., 6 h. (**e**) benzoylchloride (cat.), EtOH, 0 °C, 3 h, 17–35% (over 2 steps). (**f**) o-phenylenediamine (1.0 equiv.), EDC.HCl (1.05 equiv.), DMAP (0.5 equiv.), DCM, r.t., 6 h, 24%.

The desired DHA–HDACi hybrids were synthesized as summarized in Scheme 1. In the first step, DHA was prepared by a reduction of commercially available artemisinin. The subsequent treatment of DHA with acetic anhydride provided the key intermediate dihydroartemisinin acetate (**5**). Following the procedure of Gour et al. [27], **5** and the respective thiol or alcohol were subjected to a BF_3_-catalyzed reaction to generate the intermediates **6a–e**. The hydroxamate-based target compounds were obtained in a two-step protocol. First, an amide coupling of **6a–e** with *O*-(tetrahydro-2*H*-pyran-2-yl)hydroxylamine using EDC/DMAP as coupling agent afforded the THP-protected hydroxamic acids. Cleavage of the THP protection group was achieved by using catalytic amounts of benzoyl chloride in ethanol to provide the target compounds **7a–e**. The o-aminoanilide **8** was prepared from **6a** and *o*-phenylenediamine by another EDC/DMAP-mediated amide coupling reaction. Compounds **6a**, **7a**, **7b**, **7d**, and **8** were obtained as mixture of C-10 α- and β-isomers, whereas **7c** and **7e** were separated by preparative HPLC into the α-thiolactols ((α)-**7c** and (α)-**7e**) and the β-thiolactols ((β)-**7c** and (β)-**7e**).

### 2.2. HDAC Inhibitory Activities and Selectivity Profiles

*P. falciparum* histone deacetylase 1 (PfHDAC1) is currently the only PfHDAC available in recombinant form. However, due to the low purity of commercially available PfHDAC1 and its very low catalytic activity in the absence of endogenous cofactors, it is not suitable to be used in biochemical assays [28]. We therefore tested all synthesized DHA–HDACi hybrids for their inhibition of human HDAC1 (as representative class I isoform, hHDAC1) and HDAC6 (as representative class II isoform, hHDAC6) using ZMAL (Z-Lys(Ac)-AMC) as the substrate. Vorinostat and DHA were used as the positive and negative controls, respectively. The results from the screening are summarized in Table 1. As expected, compound **6a** featuring a carboxylic acid as a weak ZBG showed no noteworthy inhibition of hHDAC1 and hHDAC6 (IC_50_ > 10 µM). Somewhat surprisingly, the *o*-aminoanilide **8** demonstrated low HDAC inhibitory activity (hHDAC1 IC_50_ > 3.33 µM; hHDAC6 IC_50_ > 10 µM). Although this ZBG has been successfully utilized in multiple potent class I selective HDACi, such as the approved drug tucidinostat and the clinical candidate entinostat, our results indicate that the combination of the bulky DHA cap, a vinylbenzyl linker, and the *o*-aminoanilide ZBG is unsuitable to achieve potent inhibition of hHDAC1 and hHDAC6. Fortunately, all hydroxamate-based hybrids of type **7** turned out to be inhibitors of hHDAC1 and/or hHDAC6 although with different potencies and selectivity profiles. (α)-**7e** (hHDAC1 IC_50_ > 10 µM; hHDAC6 IC_50_ = 1.101 µM) and (β)-**7e** (hHDAC1 IC_50_ > 10 µM; hHDAC6 IC_50_ = 1.002 µM) displayed the weakest inhibitory activity among the hydroxamates. Interestingly, the α- and β-isomers showed almost identical HDAC inhibitory activity, thus indicating that the configuration of the thioether, which connects the DHA cap with the HDAC linker, does not affect the HDAC inhibition significantly. Compounds **7a** (selectivity index (SI^1/6^): 3) and **7b** (SI^1/6^: 10) revealed submicromolar inhibitory activity against HDAC1 and HDAC6 with a slight preference for HDAC6. In contrast, all compounds containing the benzyl linker **3** showed potent and selective inhibition of hHDAC6 over hHDAC1. Again, we did not observe a significant impact of the configuration of the thiolactol group, and (α)-**7c** (hHDAC1 IC_50_ = 2.00 µM; hHDAC6 IC_50_ = 0.036 µM; SI^1/6^: 56) and (β)-**7c** (hHDAC1 IC_50_ = 2.79 µM; hHDAC6 IC_50_ = 0.041 µM; SI^1/6^: 68) displayed comparable HDAC6 inhibition and selectivity. Notably, compound **7d** emerged as the most potent and selective HDAC6 inhibitor (hHDAC1 IC_50_ = 2.49 µM; hHDAC6 IC_50_ = 0.014 µM; SI^1/6^:178). 

**Figure 2:**
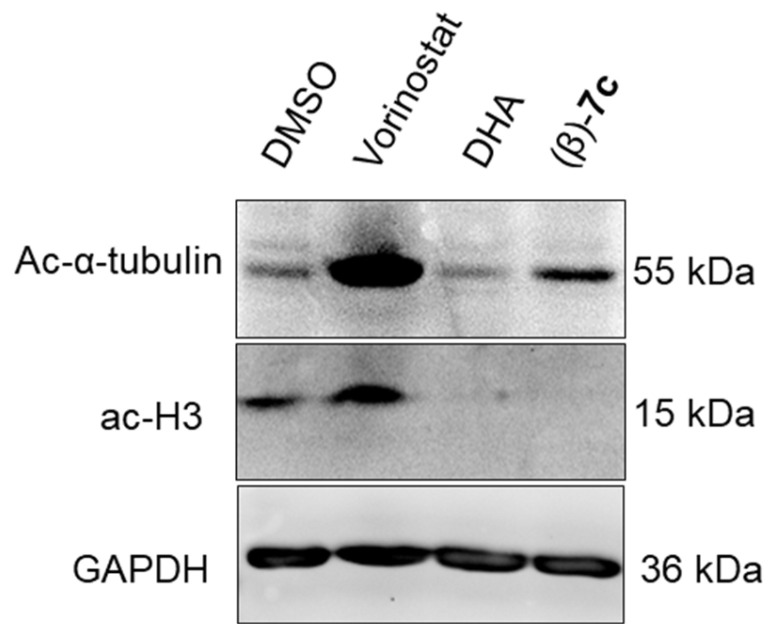
K562 cells were treated with DHA, (β)-**7c**, and vorinostat (0.4 µM) for 24 h. Subsequently, cell lysates were immunobloted with anti-acetyl-α-tubulin and acetyl-histone H3 antibodies, whereas GAPDH served as a loading control. The experiments were repeated three times (*n* = 3), and a representative blot is shown here.

In order to test cellular HDAC inhibitory activity of the DHA–HDACi, immunoblotting was performed by investigating the HDAC6 selective hybrid inhibitor (β)-**7c** in comparison to DHA and the reference panHDACi vorinostat. A representative immunoblot is shown in Figure 2. The treatment of K562 leukemic cells with the DHA–HDAC hybrid inhibitor (β)-**7c** induced acetylation of α-tubulin (marker of HDAC6 inhibition) but did not induce acetylation of histone H3 (marker of HDAC1 inhibition), which is in line with the isoform selectivity profile of (β)-**7c** (see Table 1). As expected, the panHDACi vorinostat caused hyperacetylation of α-tubulin and histone H3, whereas the negative control DHA affected the acetylation level neither of α-tubulin nor of histone H3. Comparatively weaker induction of Ac-α-tubulin expression in the case of (β)-**7c** as opposed to vorinostat might be related to differences in cellular uptake and/or stability.

### 2.3. Docking of (α)-***7c*** and (β)-***7c***

**Figure 3:**
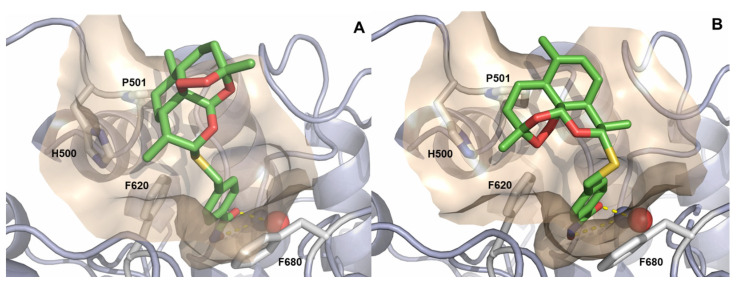
Docking pose of (α)-**7c** (**A**) and (β)-**7c** (**B**) in the catalytic domain 2 of HDAC6 (PDB: 5EDU [29]). Ligands are colored green and are depicted as sticks. The catalytic Zn^2+^-ion is shown as a gray sphere, and water is shown as a red sphere. The protein backbone is shown as light blue cartoon including the wheat-colored protein surface surrounding the ligand. The binding interactions of the hydroxamic acids are depicted as yellow, dashed lines.

Despite their different configuration at C-10 thiolactol group, (α)-**7c** and (β)-**7c** demonstrated almost identical inhibitory activity towards HDAC6. To shed light into this phenomenon, we docked (α)-**7c** and (β)-**7c** into the second catalytic domain of HDAC6 (PDB: 5EDU [29]). The experimental details are summarized in the Supplementary Materials. In the case of both compounds, the zinc-binding occurs in a monodentate manner, binding the zinc ion through the hydroxamate moiety and the Zn^2+^-bound water molecule via the carbonyl oxygen (see Figure 3). The benzylic linkers are sandwiched between two phenylalanine (**F620** and **F680**) residues, thereby stabilizing the conformations via π-π stacking. Despite their different stereochemistry at the thiolactol connecting unit, both cap groups of (α)-**7c** and (β)-**7c** are oriented into the L1-loop formed by the amino acids **H500** and **P501 [29]**. The main interactions of the cap are most likely of hydrophobic origin. In our docking models, the diasteromers (α)-**7c** and (β)-**7c** display nearly the same interactions with the HDAC6 enzyme, which further support our determined inhibitory activities against this HDAC isoform.

### 2.4. Antiplasmodial Properties and Parasite Selectivity

In order to investigate the antiplasmodial properties of the synthesized DHA–HDACi hybrids, all compounds were screened for their activity against the drug-sensitive 3D7 line of *P. falciparum* and the multidrug-resistant *P. falciparum* Dd2 line. DHA and vorinostat were included as positive controls. Selected compounds that were tested in the ring stage survival assay with the artemisinin-resistant Dd2 R539T (see Section 2.5) were also tested in the standard growth inhibition assay for comparison. The results from this screening are presented in Table 2. All DHA–HDACi hybrids demonstrated potent antiplasmodial properties with IC_50_ values in the single-digit nanomolar range. Notably, all compounds clearly outperformed the panHDACi vorinostat (Pf3D7 IC_50_ = 241.8 nM; PfDd2 IC_50_ = 424.9 nM). However, no clear structure–activity relationships could be observed. Importantly, even compounds with low (**8**; Pf3D7 IC_50_ = 2.5 nM; PfDd2 IC_50_ = 2.5 nM) or no (**6a**; Pf3D7 IC_50_ = 5.9 nM; PfDd2 IC_50_ = 5.8 nM) hHDAC inhibition demonstrated comparable antiplasmodial activity to DHA (Pf3D7 IC_50_ = 3.0 nM; PfDd2 IC_50_ = 1.8 nM) These results indicate that the antiplasmodial properties of the DHA–HDACi hybrids originate primarily from the DHA part of the molecules. 

To investigate the selectivity of the DHA–HDACi hybrids for the parasites vs. human cells, we tested all compounds for their cytotoxicity against the A2780 cell line. Again, vorinostat and DHA were used as controls. The results and selectivity indices (SIs) are summarized in Table 2. All compounds, except DHA (A2780 IC_50_ = 0.97 µM) and (β)-**7c** (A2780 IC_50_ = 0.88 µM), displayed human cell cytotoxicity in the single-digit micromolar concentration range. Six out of nine compounds tested, exhibited improved selectivity indices compared to DHA (SI^A2780/Pf3D7^: 323; SI^A2780/PfDd2^: 539). Overall, compound **7a** (SI^A2780/Pf3D7^: 750; SI^A2780/PfDd2^: 1147) and (β)-**7e** (SI^A2780/Pf3D7^: 804; SI^A2780/PfDd2^: 1306) were the most parasite-selective compound in this series.

Although the introduction of the HDACi linker and ZBG to the DHA scaffold did not lead to improved antiplasmodial activity against the 3D7 and Dd2 line of *P. falciparum*, we speculated that the dual mode of action might provide improved activity against artemisinin-resistant parasites.

### 2.5. Ring-Stage Survival Assay

**Figure 4:**
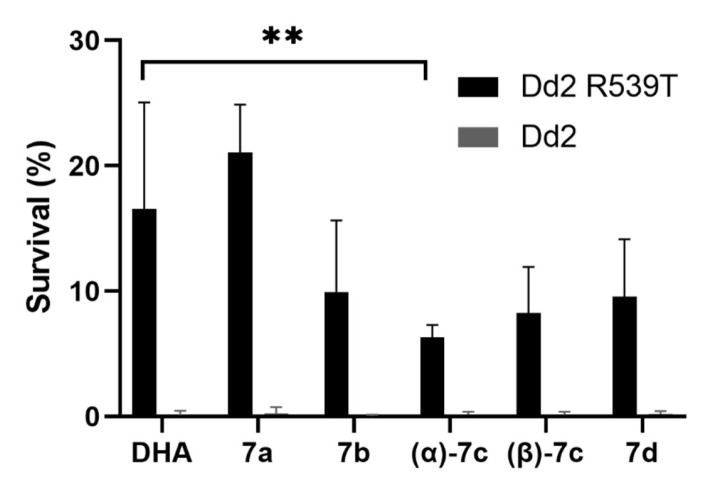
Survival of the artemisinin-resistant (Dd2 R539T) and sensitive (Dd2) *P. falciparum* line after treatment with 700 nM of the respective compound (DHA or DHA–HDACi hybrid) in the ring-stage survival assay. Results show the survival in percent of the drug-treated parasites in relation to the untreated control (DMSO) parasites. Each experiment was performed twice in duplicate. All compounds, except **7a**, were slightly more active, with (α)-**7c** being significantly more active than DHA alone (unpaired *t*-test: ** *p* < 0.005).

Artemisinin resistance in vivo in patients is defined as delayed parasite clearance after treatment with a clearance half-life longer than 5 h [30,31] that is associated with mutations in the kelch13 gene [32]. Unlike for most antimalarial drugs, this partial artemisinin resistance cannot be detected in vitro by standard growth inhibition assays, which determine the IC_50_ after an incubation period for one or several asexual replication cycles (see our results for PfDd2 R539T in Table 2). Instead, the ring-stage survival assay is used to quantify the surviving population of parasites after a short drug pulse of the ring stage of 6 h. Artemisinin-resistant parasites treated with selected DHA–HDACi hybrid molecules, except **7a**, exhibited a lower survival than those parasites treated with DHA alone (Figure 4). The lowest survival could be observed for parasites treated with (α)-**7c**. Artemisinin-sensitive Dd2 parasites treated with DHA–HDACi hybrid compounds as well as DHA had a low survival, as expected (survival ≤ 0.25%).

### 2.6. Antileukemia Properties

In addition to the outstanding antimalarial properties of artemisinin and derivatives, there is clear evidence that artemisinin and analogues possess both in vitro and in vivo activities against various types of cancer including leukemia [33]. Hence, to elucidate the antileukemia properties of the DHA–HDACi hybrids, we screened **6a**, **7a–e**, and **8** along with DHA and vorinostat as controls on a semiautomatic drug screening platform using five cell lines from different leukemia entities comprising chronic myeloid leukemia (CML), acute myeloid leukemia (AML), and acute lymphoblastic B-cell and T-cell leukemias (B-ALL and T-ALL). The results are highlighted in Table 3. Strikingly, all hydroxamate-based hybrids (**7a–e**) and the *o*-aminoanilide **8** exceeded the antiproliferative potential of DHA in four out of five cell lines (K562, HL60, NALM6, and MOLM13). Thus, in contrast to the data against the asexual blood stage *P. falciparum* parasites (Table 2), the HDACi part of the hybrid molecules appears to contribute to the antiproliferative properties of the compounds. This assumption is supported by the reduced antiproliferative activity of compound **6a**, which demonstrated negligible ability to inhibit human HDACs (Table 1). Overall, the B-ALL cell line NALM6 displayed the highest sensitivity toward the hybrid compounds, whereas the T-ALL cell line HPBALL showed the lowest sensitivity. On average across the five cell lines, hybrid **7a** demonstrated the highest antiproliferative potential with IC_50_ values ranging from 0.15 µM to 1.36 µM. Importantly, **7a** (NALM6 IC_50_ = 0.15 µM) clearly outperformed both control compounds in the NALM6 cell line with ~4-fold (vorinostat; NALM6 IC_50_ = 0.63 µM) and ~17-fold (DHA; NALM6 IC_50_ = 2.50 µM) improved antiproliferative activity.

**Figure 5:**
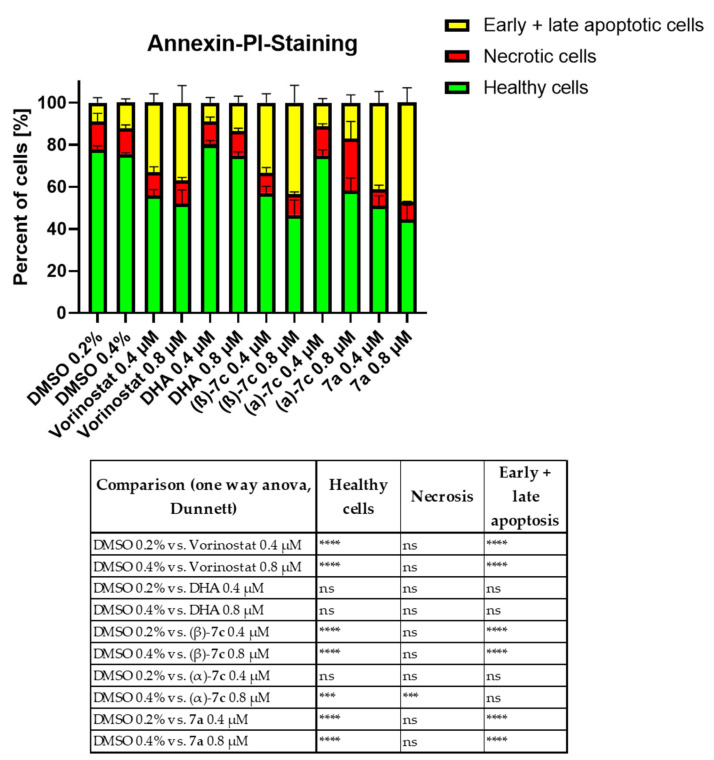
Apoptosis assay via annexin V/PI measurement after 48 h treatment of K562 cells with the respective inhibitors at the depicted concentrations. Significance was calculated using the sum of (early and late) apoptotic cells and necrotic cells vs. vehicle control (DMSO) from three independent measurements, using one-way ANOVA test (nonsignificant or ns, *** *p* < 0.0005 and **** *p* < 0.0001).

To further investigate the efficacy of the DHA–HDACi hybrids for their ability to induce apoptosis, we performed annexin V-PI measurements after treating K562 cells with the respective compound for 48 h using DHA and the reference HDACi vorinostat as controls (Figure 5). In line with the cell viability data, the exposure of K562 cells to the DHA–HDACi hybrids **7a** and (β)-**7c** significantly induced apoptosis as compared to DHA. Notably, apoptosis induction was comparable to the FDA-approved HDACi vorinostat, whereas only (α)-**7c** incubation induced significant necrosis. Taken together, the obtained data strongly suggest that the DHA–HDACi hybrids exhibit superior antileukemia activity to DHA and are even comparable with the known reference panHDACi vorinostat. Finally, to investigate the selectivity of the three most promising hybrids for leukemia cells over noncancer cells, we screened **7a**, α-**7c**, and β-**7c** for their cytotoxicity against two healthy individually-derived fibroblasts. The results are presented in Table 4. Importantly, all hybrids demonstrated clearly reduced cytotoxicity against fibroblasts compared to leukemia cells (Table 4), thereby leading to high selectivity indices (SI) of up to 87 in the case of **7a** (see Table S3, Supplementary Materials).

## 3. Materials and Methods

### 3.1. Chemistry

All chemical reagents were purchased from abcr GmbH (Karlsruhe, Germany), Acros Organics (Geel, Belgium), Carbolution (St. Ingbert, Germany), Flurochem (Hadfield, United Kingdom), Sigma-Aldrich (St. Louis, MO, USA), TCI Chemicals (Eschborn, Germany) or VWR (Langenfeld, Germany) and were used without further purification. Chemical reactions were monitored by thin-layer chromatography (TLC) using Merck F_254_ silica gel 60 plates. Analysis was performed either with a 254/365 nm UV lamp or by appropriate straining. Column chromatography was carried out on silica gel 60 (Merck, 32–63 µm). ^1^H and ^13^C NMR spectra were recorded either on a Bruker Avance III HD 400 MHz (Billerica, MA, USA) at a frequency of 400 MHz (^1^H) and 101 MHz (^13^C), a Bruker 500 DRX (Billerica, MA, USA) at a frequency of 500 MHz (^1^H) and 126 MHz (^13^C), a Varian/Agilent Mecury-plus-400 (Santa Clara, CA, USA) at a frequency of 400 MHz (^1^H) and 101 MHz (^13^C), or a Varian/Agilent Mecury-plus-300 (Santa Clara, CA, USA) at a frequency of 300 MHz (^1^H) and 75 MHz (^13^C). Chemicals shifts were reported in parts per million (ppm, δ units), and coupling constants were reported in Hertz (Hz). The splitting pattern of each signal was reported as singlet (s), doublet (d), triplet (t), quartet (q), multiplet (m), or as a combination of these. Multiplicities and coupling constants were reported as measured and might disagree with the expected values. High resolution mass spectra were obtained either with a Bruker Daltonik GmbH micrOTOF (Billerica, MA, USA) coupled to a LC Packings Ultimate HPLC system (Waltham, MA, USA) and controlled by micrOTOFControl3.4 and HyStar 3.2-LC/MS (Billerica, MA, USA) or with a Bruker Daltonik GmbH ESI-qTOF Impact II (Billerica, MA, USA) coupled to a Dionex UltiMateTM 3000 UHPLC system (Sunnyvale, CA, USA) and controlled by micrOTOFControl 4.0 and HyStar 3.2-LC/MS MS (Billerica, MA, USA). Analytical HPLC analysis was performed with a Thermo Fisher Scientific UltiMate 3000 UHPLC system (Waltham, MA, USA) with a Nucleodur 100–5 C18 (250 × 4.6 mm, Macherey Nagel, Düren, Germany) column using a flow rate of 1 mL/min at 25 °C, and the compounds were detected using UV absorption at 210 or 250 nm. For preparative purposes, a Varian ProStar system (Santa Clara, CA, USA) with a Nucleodur 5 u C18 HTec (150 × 32 mm, Macherey Nagel, Düren, Germany) column with a flow rate of 15 mL/min was used. Detection was implemented by UV absorption measurement at a wavelength of λ = 220 nm and λ =254 nm. MeCN (A) and bidest. H_2_O with 0.1% TFA (B) were used as eluents, and the gradients as described in the following Table 5 were used for both analytical and preparative purposes. The purity of all final compounds was 95% or higher. 

The following solvents, reagents, and terms were abbreviated: 1-ethyl-3-(3-dimethylaminopropyl)carbodiimide hydrochloride (EDC.HCl), 4-dimethylaminopyridine (DMAP), cyclohexane (CH), diastereomer (DS), diasteriomeric ratio (*dr*), dichloromethane (DCM), dihydroartemisinin (DHA), dihydroartemisinin acetate (DHAA), ethanol (EtOH), ethyl acetate (EA), methanol (MeOH), and *N*,*N*-dimethylformamide (DMF).

The following compounds were synthesized according to the literature: dihydroartemisinin [34] as well as dihydroartemsinin acetate [27], and all spectroscopic data were in agreement with the literature. 

### 3.2. General Method for the Synthesis of DHA-Coupled Carboxylic Acids ***6a–e***

DHAA (1.00 eq.) and the corresponding alcohol/thiol (1.20 eq.) were dissolved in dry DCM (1.00 mL/mmol). The solution was cooled to −20 °C followed by the dropwise addition of boron trifluoride etherate (1.05 eq.). The reaction was stirred until TLC showed completion of the reaction (20–30 min). Subsequently, the reaction was diluted with DCM (10 mL/mmol), and the organic phase was washed with ice-cold water (5 mL/mmol) twice. The organic phase was dried over sodium sulfate and concentrated at reduced pressure. All compounds were purified by column chromatography, except compound **6e**, which was used in the next step without further purification. 

#### 3.2.1. (E)-3-(4-((((3R,5aS,6R,8aS,9R,12R,12aR)-3,6,9-Trimethyldecahydro-12H-3,12-epoxy[1,2]dioxepino[4,3-i]isochromen-10-yl)oxy)methyl)phenyl)acrylic acid (**6a**)

Yellowish, highly viscous oil (59% yield); purified by column chromatography, eluent DCM: MeOH 98:2; *t*_R_ = 22.00 min (method B), purity = 98.7%; ^1^H-NMR (DMSO-*d*_6_) δ: 7.67 (d, 2 H, ^3^*J* = 8.2 Hz), 7.58 (d, 1 H, ^3^*J* = 16.0 Hz), 7.36 (d, 2 H, ^3^*J* = 8.1 Hz), 6.51 (d, 1 H, ^3^*J* = 16.0 Hz), 5.40 (s, 1 H), 4.82 (m, 2 H), 4.46 (d, 1 H, ^3^*J* = 12.9 Hz), 2.49–2.40 (m, 1H, under solvent), 2.19 (td, 1 H, ^3^*J* = 14.1, 3.84 Hz), 2.04–1.95 (m, 1 H), 1.85–1.64 (m, 3 H), 1.60–1.49 (m, 1 H), 1.45–1.34 (m, 2 H), 1.30 (s, 3 H), 1.15 (td, 1 H, ^3^*J* = 11.38, 6.63 Hz), 0.93–0.81 (m, 7 H) ppm; ^13^C-NMR (DMSO-*d*_6_) δ: 167.6, 143.7, 140.7, 133.3, 128.2 (2 C), 127.5 (2 C), 119.0, 103.4, 100.6, 87.1, 80.5, 79.2, 68.7, 52.1, 43.8, 36.6, 36.0, 34.1, 30.5, 25.7, 24.3, 24.1, 20.2, 12.8 ppm; HR-ESI-MS (*m*/*z*) for [M-H]^−^ calculated: 443.2070, found: 443.2060.

#### 3.2.2. 6-(((3R,5aS,6R,8aS,9R,12S,12aR)-3,6,9-Trimethyldecahydro-12H-3,12-epoxy[1,2]dioxepino[4,3-i]isochromen-10-yl)thio)hexanoic acid (**6b**)

Colorless, viscous oil (64% yield); purified by column chromatography, eluent CH: EA 2:1; ^1^H-NMR (DMSO-*d*_6_) δ: 5.46/5.37 (s, 1 H, DS1/DS2), 5.25/4.61 (d, 1 H, ^3^*J* = 5.3/10.7 Hz, DS1/DS2), 2.71–2.62 (m, 1 H), 2.56 (td, 1 H, ^3^*J* = 7.3, 3.6 Hz), 2.33 (td, 1 H, ^3^*J* = 7.4, 3.6 Hz), 2.20 (td, 3 H, ^3^*J* = 7.3, 2.6 Hz), 2.03–1.94 (m, 1 H), 1.85–1.75 (m, 1 H), 1.64–1.47 (m, 8 H), 1.41–1.31 (m, 3 H), 1.28 (s, 3 H), 1.20–1.11 (m, 1 H), 0.99–0.79 (m, 8 H) ppm; ^13^C-NMR (DMSO-*d*_6_) δ: 174.4, 103.3, 91.3, 80.2, 79.8, 51.4, 45.6, 36.3, 35.9, 33.6 (2 C), 31.8, 29.2, 28.0 (2 C), 25.6, 24.4, 24.1, 20.4, 20.1, 14.8 ppm; HR-ESI-MS (*m*/*z*) for [M+Na]^+^ calculated: 437.1974, found: 437.1978.

#### 3.2.3. 4-((((3R,5aS,6R,8aS,9R,10R,12S,12aR)-3,6,9-Trimethyldecahydro-12*H*-3,12-epoxy[1,2]dioxepino[4,3-i]isochromen-10-yl)thio)methyl)benzoic acid (**6c**)

Colorless, viscous oil (75% yield); purified by column chromatography, eluent DCM: MeOH 99.5:0.5; ^1^H-NMR (DMSO-*d*_6_) δ: 7.89 (d, 2 H, ^3^*J* = 8.2 Hz), 7.45 (d, 2 H, ^3^*J* = 8.3 Hz), 5.51 (s, 1 H), 5.20 (d, 1 H, ^3^*J* = 5.4 Hz), 3.90 (q, 2 H, *J* = 13.1 Hz), 2.80 (dt, 1 H, ^3^*J* = 7.4, 4.9 Hz), 2.19 (ddd, 1 H, ^3^*J* = 14.5, 13.2, 3.9 Hz), 2.02 (ddd, 1 H, ^3^*J* = 14.6, 4.9, 2.9 Hz), 1.86–1.75 (m, 1 H), 1.68 (dd, 1 H, ^3^*J* = 13.4, 3.6 Hz), 1.64–1.55 (m, 2 H), 1.48–1.32 (m, 3 H), 1.31 (s, 3 H), 1.20–1.12 (m, 1 H), 0.95–0.84 (m, 4 H), 0.78 (d, 3 H, ^3^*J* = 7.3 Hz) ppm; ^13^C-NMR (DMSO-*d*_6_) δ: 167.1, 143.5, 129.5 (2 C), 129.1 (2 C), 103.6, 87.3, 85.0, 80.5, 79.2, 52.1, 44.3, 36.6, 36.0, 35.2, 33.8, 31.5, 25.6, 24.2, 23.9, 20.1, 14.3 ppm; HR-ESI-MS (*m*/*z*) for [M+H]^−^ calculated: 433.1685, found: 433.1690.

#### 3.2.4. 4-((((3R,5aS,6R,8aS,12R,12aR)-3,6,9-Trimethyldecahydro-12*H*-3,12-methano[1,2]dioxepino[4,3-i]isochromen-10-yl)oxy)methyl)benzoic acid (**6d**)

Yellowish, viscous oil (55% yield); purified by column chromatography, eluent DCM: MeOH 99.5:0.5; ^1^H-NMR (DMSO-*d*_6_) δ: 12.86 (bs, 1 H), 7.93 (m, 2 H), 7.46 (m, 2 H), 5.75 (s, 1 H), 4.86 (m, 2 H), 4.55 (m, 1 H), 2.48–2.41 (m, 1 H, under DMSO-D6), 2.25–2.14 (m, 1 H), 2.03–1.96 (m, 1 H), 1.86–1.68 (m, 3 H), 1.62–1.55 (m, 1 H), 1.46 (dd, 1 H, ^3^*J* = 7.8, 4.2 Hz), 1.37 (m, 1 H), 1.30 (s, 3 H), 1.23–1.12 (m, 2 H), 0.93–0.84 (m, 7 H) ppm; ^13^C-NMR (DMSO-*d*_6_) δ: 167.1, 143.6, 129.7, 129.4/129.3 (2 C, DS2/DS1), 127.1/127.0 (2 C, DS2/DS1), 103.4, 100.7, 87.1, 80.5/80.1 (DS1/DS2), 68.6, 52.1, 43.8, 36.6, 36.0, 34.1, 30.5, 25.7/25.6 (DS2/DS1), 24.2, 24.0, 20.1/20.1 (DS1/DS2), 12.8/12.5 (DS1/DS2) ppm; HR-ESI-MS (*m*/*z*) for [M+H]^−^ calculated: 417.1913, found: 417.1899.

### 3.3. General Method for the Synthesis of the Hydroxamic Acids ***7a–e***

The respective carboxylic acid (1.00 eq.) and *O*-(tetrahydro-2*H*-pyran-2-yl)hydroxylamine (1.00 eq.) were dissolved in DCM (3 mL/0.1 mmol) and cooled to 0 °C. DMAP (0.50 eq.) and EDC.HCl (1.00 eq.) were added. The reaction mixture was allowed to warm to r.t. and stirred for 6 h. After completion of the reaction (TLC), it was diluted with DCM (2 mL/0.1 mmol) and washed with water (2 mL/0.1 mmol) twice. The organic phase was dried over sodium sulfate, and the solvent was removed under reduced pressure. The crude residue was dissolved in ethanol (1 mL/0.1 mmol) and cooled to 0 °C. One drop benzoyl chloride was added, and the mixture was stirred for 12 h at r.t. Subsequently, the solvent was removed under reduced pressure, and the crude was purified directly via preparative HPLC or column chromatography.

#### 3.3.1. (E)-*N*-Hydroxy-3-(4-((((3R,5aS,6R,8aS,9R,12R,12aR)-3,6,9-trimethyldecahydro-12*H*-3,12-epoxy[1,2]dioxepino[4,3-i]isochromen-10-yl)oxy)methyl)phenyl)acrylamide (**7a**)

Yellowish, high viscous oil (35% yield) with 2:7 *dr* (DS1:DS2;, purified by column chromatography, eluent DCM: MeOH 99:1; *t*_R_ = 32.28/33.68 min (method E), purity = 99.6%; ^1^H-NMR (CDCl_3_) δ: 7.71 (d, 1 H, ^3^*J* = 11.5 Hz), 7.48 (d, 2 H, ^3^*J* = 7.1 Hz), 7.32 (d, 2 H, ^3^*J* = 7.2 Hz), 6.39 (s, 1 H), 5.50/5.45 (s, 1 H, DS1/DS2), 5.08/4.91 (d/m, 1 H, ^3^*J* = 4.7 Hz), 4.91 (m, 1 H), 4.53 (d, 2 H, ^3^*J* = 12.9 Hz), 2.73–2.63 (m, 1 H), 2.38 (td, 1 H, ^3^*J* = 13.5, 3.6 Hz), 2.10–1.99 (m, 1 H), 1.96–1.74 (m, 3 H), 1.63 (d, 1 H, ^3^*J* = 13.7 Hz), 1.54–1.47 (m, 1 H), 1.45 (s, 3 H), 1.23 (m, 3 H), 1.02–0.75 (m, 7 H) ppm; ^13^C-NMR (DMSO-*d*_6_) δ: 162.8, 139.9, 138.1, 133.9, 127.7 (2 C), 127.5 (2 C), 118.9, 103.4/102.4 (DS2/DS1), 101.3/100.6 (DS1/DS2), 88.3/87.1 (DS1/DS2), 81.2/80.5 (DS1/DS2), 68.8, 52.1/51.4 (DS2/DS1), 45.9/43.8 (DS1/DS2), 36.7/36.4 (DS2/DS1), 36.1, 34.1/33.9 (DS2/DS1), 31.1/30.5 (DS1/DS2), 25.7/25.6 (DS2/DS1), 24.3, 24.1, 20.2/19.9 (DS2/DS1), 19.3/12.9 (DS1/DS2) ppm; HR-ESI-MS (*m*/*z*) for [M+H]^+^ calculated: 460.2335, found: 460.2343.

#### 3.3.2. *N*-Hydroxy-6-(((3R,5aS,6R,8aS,9R,12S,12aR)-3,6,9-trimethyldecahydro-12*H*-3,12-epoxy[1,2]dioxepino[4,3-i]isochromen-10-yl)thio)hexanamide (**7b**)

Colorless, high viscous oil (17% yield) with 1:4 *dr* (DS1:DS2); purified by column chromatography, eluent CHCl_3_: MeOH 99:1; *t*_R_ = 21.01/21.76 min (method A), purity = 98.4%; ^1^H-NMR (CDCl_3_) δ: 9.21 (bs, 1 H), 5.61/5.30 (s, 1 H, DS1/DS2), 5.25/4.53 (d, 1 H, ^3^*J* = 5.4/10.8 Hz, DS1/DS2), 2.75 (tt, 1 H, ^3^*J* = 13.6, 6.7 Hz), 2.63–2.49 (m, 1 H), 2.42–2.29 (1 H, m), 2.17 (t, 1 H, ^3^*J* = 7.2 Hz), 2.03 (tdd, 1 H, ^3^*J* = 14.9, 4.7, 3.1 Hz), 1.93–1.80 (m, 1 H), 1.79–1.54 (m, 7 H), 1.53–1.15 (m, 10 H), 1.10–0.97 (m, 1 H), 0.95 (d, 3 H, ^3^*J* = 6.2 Hz), 0.91 (d, 2 H, ^3^*J* = 7.3 Hz) ppm; ^13^C-NMR (CDCl_3_)δ: 171.2, 104.7/104.6 (DS2/DS1), 92.5/88.2 (DS2/DS1), 87.4/81.4 (DS1/DS2), 81.3/80.7 (DS1/DS2), 52.8/51.9 (DS1/DS2), 46.1/45.3/DS2/DS1), 37.5/37.3 (DS2/DS1), 36.5/36.4 (DS1/DS2), 34.5/34.1 (DS1/DS2), 32.9/28.9 (DS1/DS2), 32.6/32.4 (DS1/DS2), 32.2/32.0 (DS1/DS2), 29.3/29.2 (DS1/DS2), 28.0/27.7 (DS1/DS2), 26.2/26.0 (DS1/DS2), 24.8/24.7 (DS2/DS1), 24.6/24.6 (DS2/DS1), 21.4, 20.5/20.4 (DS1/DS2), 15.2/15.0 (DS2/DS1) ppm; HR-ESI-MS (*m*/*z*) for [M+H]^+^ calculated: 430.2263, found: 430.2258.

#### 3.3.3. *N*-Hydroxy-4-((((3R,5aS,6R,8aS,9R,10R,12S,12aR)-3,6,9-trimethyldecahydro-12*H*-3,12-epoxy[1,2]dioxepino[4,3-i]isochromen-10-yl)thio)methyl)benzamide ((α)-**7c**)

Diastereomers were separated by preparative HPLC, and (α)-7c was obtained as a colorless, highly viscous oil (13% yield); *t*_R_ = 31.97 min (method D), purity = 98.7%; ^1^H-NMR (DMSO-*d*_6_) δ: 11.18 (bs, 1 H), 7.70 (d, 2 H, ^3^*J* = 8.3 Hz), 7.40 (d, 2 H, ^3^*J* = 8.3 Hz), 5.52 (s, 1 H), 3.90 (d, 1 H, ^2^*J* = 13.1 Hz), 3.84 (d, 1 H, ^3^*J* = 13.0 Hz), 2.84–2.75 (m, 1 H), 2.26–2.13 (m, 1 H), 2.08–1.97 (m, 1 H), 1.85–1.77 (m, 1 H), 1.69 (dd, 1 H, ^3^*J* = 13.1, 3.5 Hz), 1.64–1.55 (m, 2 H), 1.48–1.36 (m, 2 H), 1.31 (s, 3 H), 1.17 (d, 1 H, ^3^*J* = 11.5, 6.5 Hz), 0.98–0.81 (m, 1 H), 0.89 (d, 3 H, ^3^*J* = 6.3 Hz), 0.79 (d, 3 H, ^3^*J* = 7.3 Hz) ppm; ^13^C-NMR (DMSO-*d*_6_) δ: 164.0, 141.6, 131.3, 128.9 (2 C), 127.0 (2 C), 103.6, 87.3, 85.0, 80.5, 52.1, 44.4, 36.6, 36.0, 35.2, 33.8, 31.5, 25.7, 24.2, 23.9, 20.1, 14.3 ppm; HR-ESI-MS (*m*/*z*) for [M+Na]^+^ calculated: 472.1770, found: 472.1753.

#### 3.3.4. *N*-Hydroxy-4-((((3R,5aS,6R,8aS,9R,10S,12S,12aR)-3,6,9-trimethyldecahydro-12*H*-3,12-epoxy[1,2]dioxepino[4,3-i]isochromen-10-yl)thio)methyl)benzamide ((β)-**7c**)

Diastereomers were separated by preparative HPLC, and (β)-7c was obtained as a colorless, highly viscous oil (11% yield); *t*_R_ = 29.76 min (method D), purity = 99.5%; ^1^H-NMR (DMSO-*d*_6_) δ: 11.17 (bs, 1 H), 7.69 (d, 2 H, ^3^*J* = 8.3 Hz), 7.43 (d, 2 H, ^3^*J* = 8.3 Hz), 5.41 (s, 1 H), 4.60 (d, 1 H, ^3^*J* = 10.7 Hz), 3.90 (s, 2 H), 2.42–2.31 (m, 1 H), 2.26-2.14 (m, 1 H), 2.07–1.96 (m, 1 H), 1.87–1.76 (m, 1 H), 1.63–1.56 (m, 1 H), 1.55–1.47 (m, 2 H), 1.46–1.22 (m, 3 H), 1.33 (s, 3 H), 1.15 (td, 1 H, ^3^*J* = 11.3, 6.6 Hz), 1.02–0.90 (m, 1 H), 0.89 (d, 3 H, ^3^*J* = 6.4 Hz), 0.75 (d, 3 H, ^3^*J* = 7.2 Hz) ppm; ^13^C-NMR (DMSO-*d*_6_) δ: 164.0, 142.4, 131.1, 129.0 (2 C), 127.0 (2 C), 103.5, 91.4, 80.3, 79.5, 51.3, 45.6, 36.3, 35.9, 33.4, 31.8, 31.6, 25.7, 24.3, 20.4, 20.0, 14.6 ppm; HR-ESI-MS (*m*/*z*) for [M+Na]^+^ calculated: 472.1770, found: 472.1752.

#### 3.3.5. *N*-Hydroxy-4-((((3R,5aS,6R,8aS,9R,10R,12R,12aR)-3,6,9-trimethyldecahydro-12*H*-3,12-epoxy[1,2]dioxepino[4,3-i]isochromen-10-yl)oxy)methyl)benzamide (**7d**)

Colorless, high viscous oil (28% yield); purified by column chromatography, eluent DCM: MeOH 99:1; *t*_R_ = 14.19/14.34 min (method C), purity = 95.6%; ^1^H-NMR (DMSO-*d*_6_) δ: 11.18 (bs, 1 H), 9.00 (bs, 1 H), 7.73 (d, 2 H, ^3^*J* = 7.9 Hz), 7.39 (d, 2 H, ^3^*J* = 8.0 Hz), 5.41 (s, 1 H), 4.81 (m, 2 H), 4.48 (d, 1 H, ^3^*J* = 12.9 Hz), 2.50–2.38 (m, 1 H, under DMSO-*d*_6_), 2.19 (dt, 1 H, ^3^*J* = 13.8, 3.8 Hz), 2.00 (dt, 1 H, ^3^*J* = 14.2, 4.0 Hz), 1.86–1.67 (m, 3 H), 1.60–1.50 (m, 1 H), 1.46–1.40 (m, 1 H), 1.39–1.34 (m, 1 H), 1.30 (s, 3 H), 1.25–1.06 (m, 2 H), 1.00–0.78 (m, 1 H), 0.90 (d, 3 H, ^3^*J* = 3.4 Hz), 0.88 (d, 3 H, ^3^*J* = 2.3 Hz) ppm; ^13^C-NMR (DMSO-D_6_) δ: 164.0, 141.6, 131.8, 126.9 (4 C), 103.4, 100.6, 87.1, 80.5, 68.6, 52.1, 43.8, 36.6, 36.0, 34.1, 30.5, 25.6, 24.2, 24.0, 20.1, 12.8 ppm; HR-ESI-MS (*m*/*z*) for [M+Na]^+^ calculated: 456.1998, found: 456.1980.

#### 3.3.6. *N*-Hydroxy-2-(4-(((3R,5aS,6R,8aS,9R,10R,12S,12aR)-3,6,9-trimethyldecahydro-12*H*-3,12-epoxy[1,2]dioxepino[4,3-i]isochromen-10-yl)thio)phenyl)acetamide ((α)-**7e**)

Diastereomers were separated by preparative HPLC, and (α)-7e was obtained as a colorless, highly viscous oil (4% yield); *t*_R_ = 14.99 min (method C), purity = 95.4%; ^1^H-NMR (DMSO-*d*_6_) δ: 10.65 (s, 1 H), 8.81 (s, 1 H), 7.42 (dd, 2 H, ^3^*J* = 8.3 Hz, ^4^*J* = 2.9 Hz), 7.21 (dd, 2 H, *J* = 13.2 Hz, ^3^*J* = 8.3 Hz), 5.60 (s, 1 H), 5.56 (d, 1 H, ^3^*J* = 5.3 Hz), 3.27 (s, 2 H), 2.90 (q, 1 H, ^3^*J* = 5.6 Hz), 2.21–2.12 (m, 1 H), 2.06–1.95 (m, 1 H), 1.88–1.77 (m, 1 H), 1.70 (d, 1 H, ^3^*J* = 9.4 Hz), 1.65–1.59 (m, 1 H), 1.51 (dd, 2 H, ^3^*J* = 12.8, 4.9 Hz), 1.45–1.41 (m, 1 H), 1.40–1.36 (m, 1 H), 1.27 (s, 3 H), 1.25-1.10 (m, 2 H), 1.04-0.88 (m, 6 H) ppm. HR-ESI-MS (*m*/*z*) for [M+Na]^+^ calculated: 472.1769, found: 472.1749.

#### 3.3.7. *N*-Hydroxy-2-(4-(((3R,5aS,6R,8aS,9R,10S,12S,12aR)-3,6,9-trimethyldecahydro-12*H*-3,12-epoxy[1,2]dioxepino[4,3-i]isochromen-10-yl)thio)phenyl)acetamide ((β)-**7e**)

Diastereomers were separated by preparative HPLC, and (β)-7e was obtained as a colorless, highly viscous oil (18% yield); *t*_R_ = 19.58 min (method B), purity = 97.3%; ^1^H-NMR (DMSO-*d*_6_) δ: 7.53 (d, 2 H, ^3^*J* = 8.3 Hz), 7.20 (d, 2 H, ^3^*J* = 8.3 Hz), 5.47 (s, 1 H), 4.90 (d, 1 H, ^3^*J* = 10.7 Hz), 3.26 (s, 2 H), 2.41–2.31 (m, 1 H), 2.24–2.13 (m, 1 H), 2.04–1.95 (m, 1 H), 1.87–1.76 (m, 1 H), 1.65–1.61 (m, 1 H), 1.61–1.53 (m, 2 H), 1.52–1.45 (m, 1 H), 1.43–1.28 (m, 2 H), 1.33 (s, 3 H), 1.19–1.09 (m, 1 H), 1.01–0.92 (m, 1 H), 0.89 (d, 3 H, ^3^*J* = 6.4 Hz), 0.81 (d, 3 H, ^3^*J* = 7.1 Hz) ppm; ^13^C-NMR (DMSO-*d*_6_) δ: 166.8, 135.0, 131.1 (2 C), 130.7, 129.3 (2 C), 103.5, 91.3, 81.9, 80.2, 51.3, 45.5, 39.0, 36.3, 35.9, 33.6, 30.9, 25.7, 24.4, 20.4, 20.0, 14.7 ppm; HR-ESI-MS (*m*/*z*) for [M+Na]^+^ calculated: 472.1769, found: 472.1753.

### 3.4. (E)-N-(2-Aminophenyl)-3-(4-((((3R,5aS,6R,8aS,9R,12R,12aR)-3,6,9-trimethyldecahydro-12H-3,12-epoxy[1,2]dioxepino[4,3-i]isochromen-10-yl)oxy)methyl)phenyl)acrylamide (***8***)

A total of 150 mg (0.29 mmol, 1.00 eq.) 6a, 17.5 mg (0.14 mmol, 0.50 eq.) DMAP, and 56.1 mg (0.29 mmol, 1.00 eq.) EDC.HCl were dissolved in dry DCM (10 mL), and the mixture was cooled to 0 °C. 93.5 mg (0.86 mmol, 3.00 eq.). *o*-phenylenediamine was dissolved in DCM (10 mL), added to the reaction mixture at once, and the resulting mixture was stirred for 3 h at r.t. Subsequently, the reaction was diluted with DCM (5 mL) and washed with water (10 mL). The aqueous phase was extracted with DCM (10 mL) twice. The combined organic extracts were dried over sodium sulfate, and the solvent was evaporated under reduced pressure. The crude product was purified by column chromatography using CHCl_3_: MeOH 99:1 to yield 34 mg (24%) of 8 as a yellowish oil with a 1:2 *dr* (DS1:DS2) (determined by ^1^H-NMR); *t*_R_ = 22.54/22.87 min (method A), purity = 96.5%; ^1^H-NMR (CDCl_3_) δ: 7.71 (d, 2 H, C-H, N-H, ^3^*J* = 15.0 Hz), 7.48 (d, 2 H, ^3^*J* = 7.5 Hz), 7.35 (d, 1 H, ^3^*J* = 7.7 Hz), 7.32–7.27 (m, 2 H), 7.05 (t, 1 H, ^3^*J* = 7.1 Hz), 6.84–6.75 (m, 2 H), 6.62 (d, 1 H, ^3^*J* = 15.6 Hz), 5.50/5.46 (s, 1 H, DS1/DS2), 5.09/4.89 (d/m, 1 H, ^3^*J* = 4.7 Hz, DS1/DS2), 4.89 (m, 1 H), 4.60/4,52 (d, 1 H, *J* = 12.5/12.8 Hz, DS1/DS2), 2.68 (dt, 1 H, ^3^*J* = 7.7, 4.2 Hz), 2.44–2.25 (m, 1 H), 2.10–1.98 (m, 1 H), 1.93–1.85 (m, 1 H), 1.84–1.78 (m, 1 H), 1.77–1.66 (m, 1 H), 1.65–1.58 (m, 1 H), 1.58–1.49 (m, 1 H), 1.45 (s, 3 H), 1.31–1.24 (m, 2 H), 1.19 (d, 1 H, ^3^*J* = 7.3 Hz), 1.04–0.85 (m, 7 H) ppm; ^13^C-NMR (CDCl_3_) δ: 164.6, 142.2, 140.8, 140.6/140.3 (DS2/DS1), 134.0/133.9 (DS1/DS2), 128.2 (3 C), 127.7 (2 C), 127.3, 125.3, 124.7, 120.2, 119.8, 118.4, 104.4/103.3 (DS2/DS1), 102.5/101.6 (DS1/DS2), 89.3/88.2 (DS1/DS2), 81.8/81.3 (DS1/DS2), 69.9/69.5 (DS1/DS2), 52.7/52.0 (DS2/DS1), 46.6/44.5 (DS1/DS2), 37.6/37.4 (DS2/DS1), 36.6/36.5 (DS1/DS2), 34.7/34.5 (DS1/DS2), 31.8/31.1 (DS1/DS2), 26.3/26.1 (DS2/DS1), 24.8/24.7 (DS2/DS1), 20.5/20.2 (DS2/DS1), 19.7/13.2 (DS1/DS2) ppm; HR-ESI-MS (*m*/*z*) for [M+H]^+^ calculated: 535.2808, found: 535.2814.

### 3.5. Biological Evaluation

#### 3.5.1. In Vitro Human HDAC1 and 6 Assay

The inhibition of human HDAC1 and 6 was determined as previously described [35]. OptiPlate-96 black microplates (Perkin Elmer Inc., Waltham, MA, USA) were used with an assay volume of 50 µL. A total of 5 µL test compound or control, diluted in assay buffer (50 mM Tris-HCl, pH 8.0, 137 mM NaCl, 2.7 mM KCl, 1 mM MgCl_2_, 0.1 mg/mL BSA), was incubated with 35 µL of the fluorogenic substrate ZMAL (Z-Lys(Ac)-AMC) (21.43 µM in assay buffer) and 10 µL of human recombinant HDAC1 (BPS Bioscience Inc., San Diego, CA, USA, Catalog# 50051) or HDAC6 (BPS Bioscience Inc., San Diego, CA, USA, Catalog# 50006) at 37 °C. After an incubation time of 90 min, 50 µL of 0.4 mg/mL trypsin in trypsin buffer (50 mM Tris-HCl, pH 8.0, and 100 mM NaCl) was added, followed by further incubation at 37 °C for 30 min. Fluorescence was measured with an excitation wavelength of 355 nm and an emission wavelength of 460 nm using a Fluoroskan Ascent microplate reader (Thermo Scientific, Waltham, MA, USA). All compounds were tested at least twice and in duplicates, and the 50% inhibitory concentration (IC_50_) was determined by plotting dose response curves and nonlinear regression with GraphPad Prism (San Diego, CA, USA).

#### 3.5.2. MTT Cell Viability Assay

The human ovarian carcinoma cell line A2780 (ECACC No. 93112519) was purchased from the European Collection of Cell Cultures (ECACC, UK) and cultured in RPMI 1640 medium supplemented with 10% fetal bovine serum, 2 mM L-Glutamine, 50 U/mL penicillin, and 50 µg/mL streptomycin. Reagents were obtained from PAN Biotech, Aidenbach Germany. The cell line was cultured at 37 °C under humidified air with 5% CO_2_ to a confluence of 80–90% before being used for further assays.

Intrinsic cytotoxicity of test compounds was determined by an MTT assay as previously described [36]. A2780 cells were seeded in 96-well plates (Starlab GmbH, Hamburg, Germany) at a density of 20,000 cells/well and allowed to attach for 12 h. Cells were then treated with increasing concentrations of the test compounds. After 72 h, MTT (3-(4,5-dimethylthiazol-2-yl)-2,5-diphenyltetrazolium bromide) (BioChemica, Applichem GmbH, Darmstadt, Germany) solution (5 mg/mL in phosphate-buffered saline) was added to determine cell survival. The formazan dye was dissolved in DMSO (Sigma-Aldrich, Darmstadt, Germany) after 1 h. Absorbance was measured at 570 nm and 690 nm in a Multiskan microplate photometer (Thermo Fisher Scientific, Waltham, MA, USA). Concentration-response curves were generated using the four-parameter logistic equation (GraphPad Prism 9.0, San Diego, USA), and mean IC_50_ values were calculated in triplicates based on at least three different experiments.

#### 3.5.3. Cell Culture (Leukemia Cell Lines and Fibroblasts)

K562 (CML), HL60 (AML), MOLM13 (AML), NALM6 (B-ALL), and HPBALL (T-ALL) cell lines (DMSZ, Braunschweig, Germany) were cultured in RPMI1640 supplemented with 10% or 15% FCS at 37 °C with 5% CO_2_. Healthy individually-derived fibroblasts were cultured in DMEM supplemented with 10% FCS. Regular cell line authentication was performed short tandem repeat (STR) analysis (DNA fingerprinting), followed by comparison with the original DSMZ cell line database.

#### 3.5.4. CellTiter-Glo Based Cell Viability Assay

Cell viability assay was performed with leukemic cell lines after 72 h incubation with the respective inhibitors using ATP-based CellTiter-Glo luminescent assay (Promega, Madison, WI, USA). Briefly, inhibitors were dispensed on 384-well white plates (Corning, NY, USA) with increasing concentrations of the inhibitors in 8 dilution steps (50 nM–25 µM), in an automated fashion, using a digital dispenser (D300e, Tecan, Switzerland). Final readouts were performed using microplate reader (Spark, Tecan), and the average 50% inhibitory concentration (IC50) for the compounds was calculated (*n* = 3) by plotting the sigmoid dose-response curve and nonlinear regression (GraphPad Prism 8, San Diego, CA, USA).

#### 3.5.5. Annexin V-PI Staining

To evaluate the induction of apoptosis upon respective inhibitor treatment, K562 cells were incubated (48 h) with inhibitors (0.4 µM or 0.8 µM) or with vehicle control (DMSO). The induction of apoptosis/necrosis was analyzed by measuring hypodiploid DNA content, mitochondrial potential, and exposure of phosphatidylserine after annexin/propidium iodide (PI) staining kit (BD, Franklin lakes, NJ, USA) using FACS.

#### 3.5.6. Immunoblotting

K562 cells were treated with the indicated concentration of the compounds or vehicle control (DMSO) for 24 h. The recovered cell pellets were lysed with RIPA buffer (50 mM Tris-HCl pH 8.0, 1% Triton X-100, 0.5% sodium deoxycholate, 0.1% SDS, 150 mM sodium chloride, and 2 mM EDTA), supplemented with protease and phosphatase inhibitors (Thermofischer Scientific, Waltham, MA, USA). An equal amount of total protein (20 µg) was resolved by SDS-PAGE and followed by transfer on nitrocellulose membrane (Sigma-Aldrich). PageRuler prestained protein ladder, 10 kDa to 180 kDa (Thermofisher Scientific, Waltham, MA, USA) was used as a protein molecular weight marker. After blocking (BSA), blots were incubated with acetylated-α-tubulin (#5335), acetylated-histone H3 (#9677), and GAPDH (#97166) antibodies (Cell Signaling Technology, Danvers, USA).

#### 3.5.7. In Vitro *P. falciparum* Growth Inhibition Assay

Antiplasmodial activity of DHA–HDACi hybrids was evaluated against the asexual stages of the laboratory *P. falciparum* strains (3D7, chloroquine-sensitive strain, and Dd2, chloroquine-resistant strain, and Dd2 R539T (MRA-1255), provided by BEI Resources carrying a kelch13-mutation that conveys partial artemisinin resistance [37]), using the histidine-rich protein 2 (HRP2) assay, as previously described. [38,39] In brief: 96-well plates were precoated with the tested compounds in a three-fold serial dilution before ring-stage parasites were added in complete culture medium at a hematocrit of 1.5% and a parasitaemia of 0.05%. After three days of incubation at 37 °C, 5% CO_2_ and 5% O_2_ plates were frozen until analyzed by HRP2-ELISA. All compounds were evaluated in duplicate in at least two independent experiments. The IC_50_ was determined by nonlinear regression analysis of log concentration-response curves using the drc-package v3.0.1 [40] of R v4.0.5. [41].

#### 3.5.8. Ring-Stage Survival Assay (0–3 h)

The effect of DHA–HDACi hybrids on artemisinin-resistant asexual stages of *P. falciparum* was assessed using the ring-stage survival assay as previously described with minor modifications [42,43]. Briefly, mature schizonts of the artemisinin-sensitive strain Dd2 and the partial artemisinin-resistant strain Dd2 R539Twere isolated using magnetic column separation. The schizonts were then put with 10 mL complete medium and 200 µL 0^+^ erythrocytes in a T-25 cell culture flask and incubated for exactly 3 h (37 °C, 5% O_2,_ and 5% CO_2_). Next, parasites were synchronized with 5% (*w*/*v*) D-sorbitol solution yielding a culture containing only newly-invaded (0–3 h) ring-stage parasites.

Ring-stage parasites were added at 2% hematocrit and 0.5% parasitemia to a 48-well plate precoated with complete medium containing either DHA (Sigma-Aldrich) (positive control) or the tested DHA–HDACi hybrids at a final concentration of 700 nM or DMSO (negative control). The plates were incubated for 6 h (37 °C, 5% O_2,_ and 5% CO_2_) after which the drugs were removed by washing 3 times with 5 mL complete medium. The pellets were then taken up in 1 mL of fresh complete medium, added to new wells of the 48-well plate, and incubated for 66 h (37 °C, 5% O_2,_ and 5% CO_2_). Parasitemia was assessed by Giemsa-stained thin blood smears by two independent readers (10,000 erythrocytes per slide). If the results were discordant by more than 50%, a third independent read was conducted. The survival was calculated as the percentage of treated versus the untreated controls (DMSO). Each assay contained two technical replicates per condition and was repeated twice; experiments for DHA were repeated 4 times in duplicate. Means and standard deviations were calculated using Microsoft Excel. The figure and comparison of survival between compounds (unpaired *t*-test) was done with GraphPad Prism 8, San Diego, CA, USA).

## 4. Conclusions

In conclusion, we designed a mini library of DHA–HDACi hybrids, and the target compounds were synthesized from commercially available artemisinin using straightforward and efficient four- or five-step protocols. The subsequent screening against human HDAC1 and HDAC6 demonstrated that all hydroxamate-based hybrids possess HDAC inhibiting properties, albeit with differences in potency and selectivity. Compounds containing the benzyl linker **3** turned out to be potent and selective HDAC6i ((α)-**7c**, (β)-**7c**, and **7d**), whereas compounds with a panobinostat-type vinylbenzyl linker (**7a**) or vorinostat-like alkyl (**7b**) linker were less selective. Interestingly, the configuration at the C-10 position had little impact on the HDAC inhibitory capacity of the compounds. The subsequent screening of this series of hybrids against drug sensitive (3D7) and resistant (Dd2) lines of *P. falciparum* revealed in all cases potent antiplasmodial properties with IC_50_ values ranging in the single-digit nanomolar concentration range. Although the compounds did not exhibit improved antiplasmodial properties against both *P. falciparum* lines (compared to DHA), (α)-**7c** showed superior activity against artemisinin-resistant parasites in a ring-stage survival assay. 

In addition to their well-established antiplasmodial properties, artemisinin and derivatives possess encouraging activities against various types of cancer including leukemia. Consequently, we screened all synthesized hybrids against a panel of five cell lines from different leukemia entities. In particular, compounds **7a**, (α)-**7c**, and (β)-**7c** displayed outstanding antiproliferative properties clearly outperforming DHA in four out of five ((β)-**7c**) and in the case of **7a** and (α)-**7c** even in all five cell lines. 

Taken together, the DHA–HDACi hybrids disclosed in the present study are promising lead structures for the development of new therapeutic modalities to treat malaria as well as leukemia and might represent a new approach to tackle artemisinin resistance.

## Figures and Tables

**Table 1:** Inhibitory activities of DHA–HDACi hybrids against human HDAC1 (hHDAC1) and HDAC6 (hHDAC6) *^a^*.

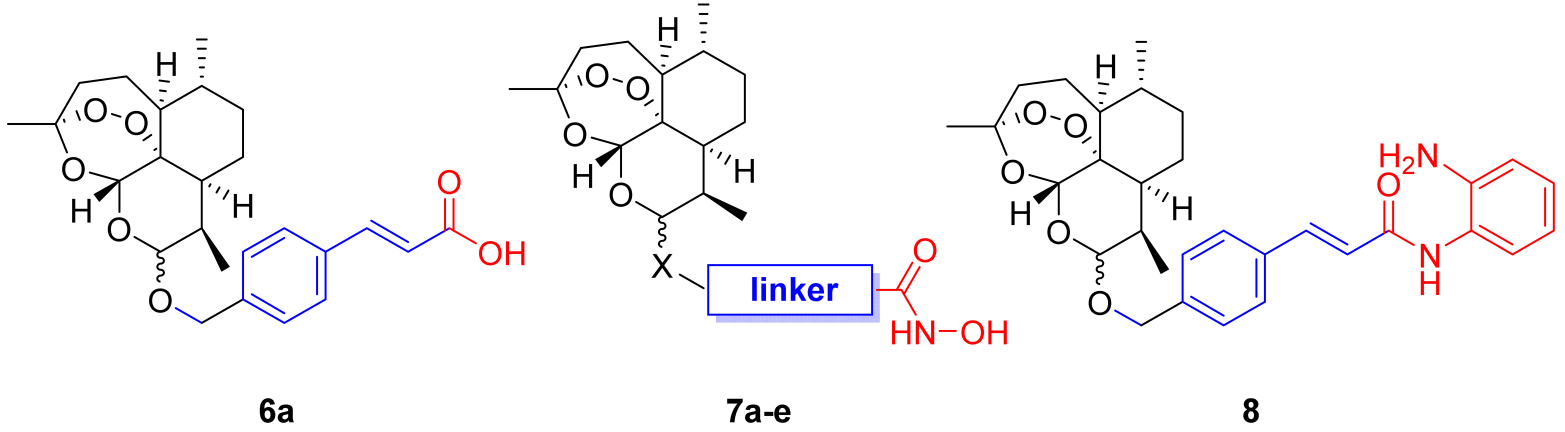					
Compound	X	Linker	hHDAC1 IC_50_ [µM] *^b^*	hHDAC6 IC_50_ [µM] *^b^*	SI^1/6^
**6a**	O	**1**	>10 *^c^*	>10 *^c^*	/
**7a**	O	**1**	0.546 ± 0.0003	0.187 ± 0.004	3
**7b**	S	**2**	0.430 ± 0.046	0.045 ± 0.005	10
(α)-**7c**	S	**3**	2.00 ± 0.200	0.036 ± 0.008	56
(β)-**7c**	S	**3**	2.79 ± 0.14	0.041 ± 0.009	68
**7d**	O	**3**	2.49 ± 0.240	0.014 ± 0.002	178
(α)-**7e**	S	**4**	>10 *^c^*	1.101 ± 0.075	>9
(β)-**7e**	S	**4**	>10 *^c^*	1.002 ± 0.130	>10
**8**	O	**1**	41% @3.33 µM *^d^*	>10 *^c^*	/
**DHA**	-	-	>10 *^c^*	>10 *^c^*	/
**Vorinostat**	-	-	0.107 ± 0.013	0.032 ± 0.008	3

*^a^* For definitions of 1, 2, 3, and 4, see Figure 1. *^b^* Assays carried out with *n* ≥ 2 (each in duplicate wells), values are shown as means ± SD. *^c^* < 50% inhibition at 10 µM. *^d^* % inhibition at 3.33 µM. Selectivity Index (SI) = (hHDAC1 IC_50_)/(hHDAC6 IC_50_).

**Table 2:** In vitro activity against asexual blood stages of *P. falciparum* parasites, cytotoxicity, and selectivity indices of **6a**, **7a–e**, and **8** *^a^*.

Compound	X	Linker	*Pf*3D7 IC_50_ [nM] *^b^*	*Pf*Dd2 IC_50_ [nM] *^b^*	*Pf*Dd2 R539T IC_50_ [nM] *^b^*	A2780 IC_50_ [µM] *^c^*	SI^A2780/*Pf* 3D7^	SI^A2780/*Pf* Dd2^
**6a**	O	**1**	5.9 ± 0.70	5.8 ± 2.8	n.d.	3.38 ± 0.21	573	583
**7a**	O	**1**	2.6 ± 1.4	1.7 ± 0.92	1.9 ± 1.4	1.95 ± 0.52	750	1147
**7b**	S	**2**	5.2 ± 0.4	6.6 ± 2.5	3.0 ± 1.2	1.52 ± 0.05	292	230
(α)-**7c**	S	**3**	3.3 ± 1.4	3.4 ± 0.12	3.3 ± 2.2	1.10 ± 0.25	333	324
(β)-**7c**	S	**3**	3.6 ± 1.1	3.2 ± 0.02	2.6 ± 2.0	0.88 ± 0.13	244	275
**7d**	O	**3**	2.8 ± 1.3	2.0 ± 0.7	1.2 ± 0.8	1.31 ± 0.32	468	655
(α)-**7e**	S	**4**	3.1 ± 0.7	1.8 ± 0.8	n.d.	1.83 ± 0.10	590	1017
(β)-**7e**	S	**4**	2.6 ± 1.3	1.6 ± 0.8	n.d.	2.09 ± 0.42	804	1306
**8**	O	**1**	2.5 ± 1.4	2.5 ± 0.14	n.d.	1.47 ± 0.98	588	588
**DHA**	-	-	3.0 ± 2.7	1.8 ± 0.02	n.d.	0.97 ± 0.07	323	539
**Vorinostat**	-	-	241.8 ± 33.4	424.9 ± 1.3	n.d.	1.09 ± 0.41	5	3

*^a^* For definitions of 1, 2, 3, and 4, see Figure 1. *^b^* Assays carried out with *n* ≥ 2 (each in duplicate wells), values are shown as means ± SD. *^c^* Assays carried out with *n* ≥ 3 (each in triplicate wells), values are shown as means ± SD. Selectivity Index (SI) = (A2780 IC_50_)/(*P. falciparum* IC_50_). n.d., not determined.

**Table 3:** IC_50_ Values of DHA–HDACi hybrids for different leukemia entities *^a^*.

Compound	X	Linker	K562 *^b^* IC_50_ [µM]	HL60 *^c^* IC_50_ [µM]	NALM6 *^d^* IC_50_ [µM]	HPBALL *^e^* IC_50_ [µM]	MOLM13 *^c^* IC_50_ [µM]
**6a**	O	**1**	4.01 ± 0.60	2.53 ± 0.56	1.31 ± 0.11	23.86 ± 4.69	0.68 ± 0.09
**7a**	O	**1**	0.31 ± 0.06	1.33 ± 0.14	0.15 ± 0.01	1.36 ± 0.03	0.22 ± 0.04
**7b**	S	**2**	0.91 ± 0.32	2.07 ± 0.03	0.26 ± 0.01	2.41 ± 0.38	0.58 ± 0.19
(α)-**7c**	S	**3**	0.62 ± 0.06	1.02 ± 0.27	0.28 ± 0.05	6.72 ± 0.44	0.33 ± 0.11
(β)-**7c**	S	**3**	0.41 ± 0.11	2.34 ± 0.46	0.25 ± 0.01	12.33 ± 0.57	0.38 ± 0.07
**7d**	O	**3**	1.00 ± 0.36	2.41 ± 0.45	0.36 ± 0.05	6.81 ± 0.54	0.39 ± 0.05
(α)-**7e**	S	**4**	0.77 ± 0.24	1.62 ± 0.12	0.44 ± 0.01	3.34 ± 0.37	0.45 ± 0.18
(β)-**7e**	S	**4**	1.71 ± 0.33	2,76 ± 0.62	0.77 ± 0.08	20.57 ± 6.13	0.64 ± 0.17
**8**	O	**1**	0.69 ± 0.11	1.64 ± 0.17	0.34 ± 0.03	1.69 ± 0.19	0.49 ± 0.08
**DHA**	-	-	2.40 ± 0.66	3.57 ± 1.04	2.50 ± 0.16	9.93 ± 1.51	0.69 ± 0.17
**Vorinostat**	-	-	0.28 ± 0.15	0.22 ± 0.05	0.63 ± 0.06	0.59 ± 0.05	0.20 ± 0.07

*^a^* For definitions of 1, 2, 3, and 4, see Figure 1. *^b^* Chronic myeloid leukemia (CML). *^c^* Acute myeloid leukemia (AML). *^d^* B-cell precursor acute lymphoblastic leukemia (B-ALL). *^e^* T-cell acute lymphoblastic leukemia (T-ALL).

**Table 4:** IC_50_ Values of DHA–HDACi hybrids for two healthy fibroblast cells.

Compound	Healthy Fibroblast 1	Healthy Fibroblast 2
	IC_50_ [µM]	IC_50_ [µM]
**7a**	13.10 ± 0.10	6.34 ± 0.79
(α)-**7c**	12.69 ± 0.12	7.41 ± 1.40
(β)-**7c**	14.67 ± 3.42	17.75 ± 6.04
DHA	>25	>25
Vorinostat	4.55 ± 0.80	3.23 ± 0.66

**Table 5:** UHPLC and preparative HPLC gradients.

Method A		Method B		Method C		Method D		Method E	
t_R_ [min]	A [%]	t_R_ [min]	A [%]	t_R_ [min]	A [%]	t_R_ [min]	A [%]	t_R_ [min]	A [%]
0	5	0	5	0	5	0	5	0	5
5	5	5	5	5	5	5	5	5	5
15	60	20	95	12	95	17	50	17	50
35	60	25	95	22	95	37	50	45	50
37	5	26	5	23	5	39	5	47	5
42	5	31	5	28	5	44	5	52	5

## Data Availability

Data is contained within the article or Supplementary Material.

## Supplementary Materials

The following supporting information can be downloaded at: https://www.mdpi.com/article/10.3390/ph15030333/s1. Table S1. Representative concentration-effect curves for key compounds; Table S2. Calculated LogP values, molecular weight, number of hydrogen bond donors and acceptors for the synthesized DHA-HDACi hybrids; Table S3. Selectivity indices range against healthy fibroblasts), docking protocols, ^1^H and ^13^C NMR spectra of final compounds.
