# Supplementary material for: Synthesis, Antiplasmodial, and Antileukemia Activity of Dihydroartemisinin–HDAC Inhibitor Hybrids as Multitarget Drugs
Source: Pharmaceuticals (Basel). 2022 Mar 9;15(3):333. doi: 10.3390/ph15030333 (PMC8952208; doi:10.3390/ph15030333)
Supplement: Supplementary file 1 [file pharmaceuticals-15-00333-s001.zip › pharmaceuticals-1511123-supplementary.pdf]

Electronic Supplementary Information

Synthesis, antiplasmodial and antileukemia activity of dihydroartemisinin-HDAC inhibitor hybrids as multi-target drugs

*Lukas von Bredow[1], Thomas Martin Schäfer[2], Julian Hogenkamp[3], Maik Tretbar[1], Daniel Stopper[4], Fabian B. Kraft[4], Julian Schliehe-Diecks[3], Andrea Schöler[1], Arndt Borkhardt[3], Sanil Bhatia[3], Jana Held[2,5], Finn K. Hansen[4]**

1 Institute for Drug Discovery, Medical Faculty, Leipzig University, Brüderstraße 34, D-04103 Leipzig, Germany
2 Institut für Tropenmedizin, Eberhard Karls Universität Tübingen, Wilhelmstr. 27, 72074, Tübingen, Germany
3 Department of Pediatric Oncology, Hematology and Clinical Immunology, Medical Faculty, Heinrich-Heine University Düsseldorf, Düsseldorf, Germany
4 Pharmaceutical Institute, Department of Pharmaceutical and Cell Biological Chemistry, University of Bonn, An der Immenburg 4, D-53121 Bonn, Germany
5 German Center for Infection Research (DZIF), partner site Tübingen, Tübingen, Germany

*Correspondence: finn.hansen@uni-bonn.de

Table of contents

1. Supplemental Tables……………………………………………………………..S3
2. Docking studies……………………………………………………………………..S6
3. ^{1}H and ^{13}C NMR spectra……………………………………………..…………S10
4. References…………………………………………………………………………..S33

1. Supplemental Tables

Table S1. Representative concentration-effect curves for key compounds.

Compound	Dose-Response Curves HDAC1	Dose-Response Curves HDAC6
**7a**	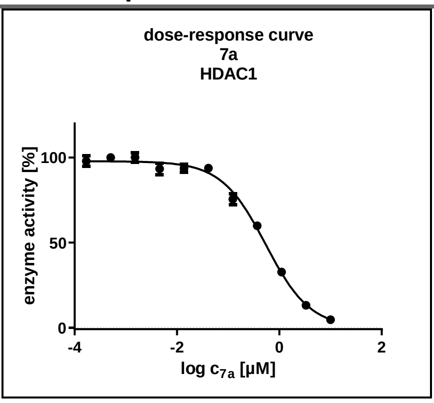	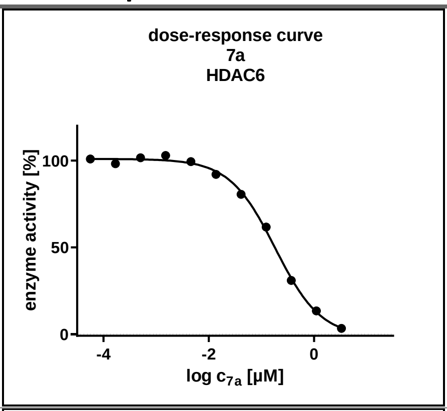
**(α)-7c**	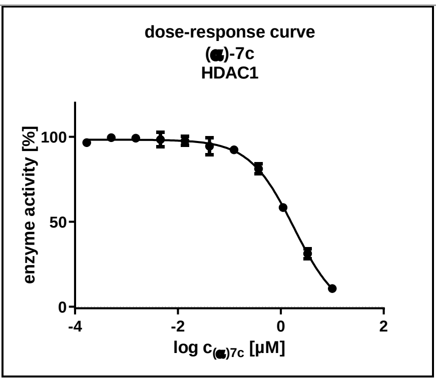	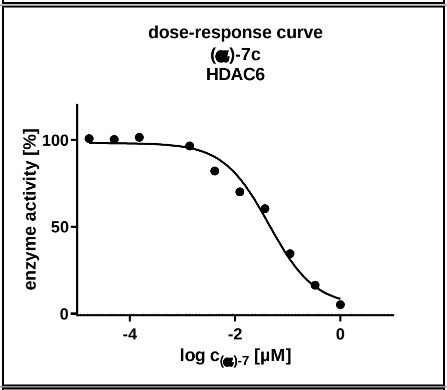
**(β)-7c**	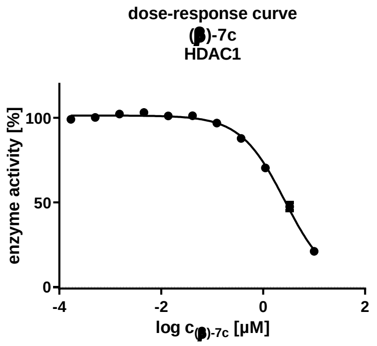	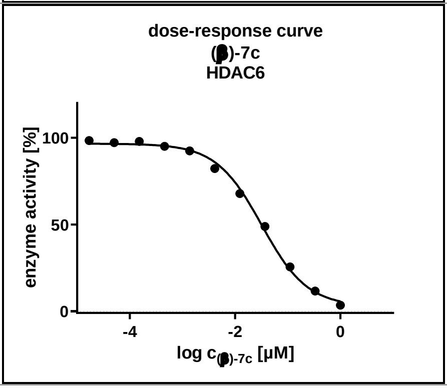

Table S2. Calculated LogP values, molecular weight, number of hydrogen bond donors and acceptors for the synthesized DHA-HDACi hybrids.

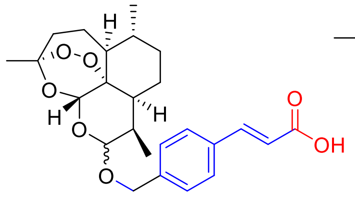

6a

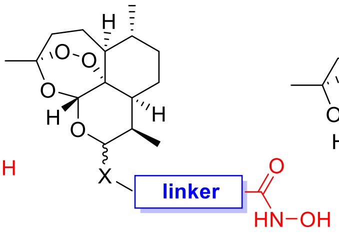

7a-e

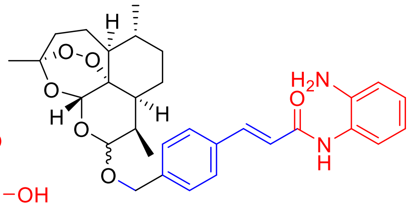

8

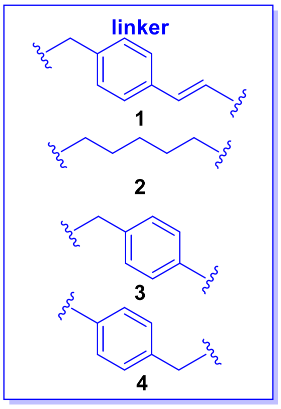

Compound	X	Linker	LogP[1]	Molecular Weight	H-Bond Donors[2]	H-Bond Acceptors[2]
**6a**	O	**1**	5.14	444.52	1	6
**7a**	O	**1**	4.65	459.54	2	7
**7b**	S	**2**	4.42	429.57	2	6
(α)-**7c**	S	**3**	5.03	449.56	2	6
(β)-**7c**	S	**3**	5.03	449.56	2	6
**7d**	O	**3**	4.31	433.50	2	7
(α)-**7e**	S	**4**	4.91	449.56	2	6
(β)-**7e**	S	**4**	4.91	449.56	2	6
**8**	O	**1**	5.58	534.65	2	7
**DHA**	-	-	3.15	284.35	1	5
**Vorinostat**	-	-	1.79	264,33	3	3

[1] Calculated by ChemDraw Professional v19.1
[2] Calculated by ChemOffice 19 MS Excel Plugin

Table S3. Selectivity indices range against healthy fibroblasts as compared to leukemia cells. Selectivity Index (SI) = (healthy fibroblast IC_{50})/(leukemia cell line IC_{50}). See Table 4 for fibroblast IC_{50} values.

**Compound**	K562	HL60	NALM6	HPB ALL	MOLM13
**7a**	21-42	5-10	42-87	5-10	29-60
**(α)-7c**	12-21	7-12	27-45	1.1-1.9	23-39
**(β)-7c**	36-43	6-8	59-71	1.2-1.4	39-47
**Vorinostat**	12-16	15-21	5-7	6-8	16-22

2. Docking of (α)-7c and (β)-7c

The crystal structure of human HDAC6 (PDB: 5EDU [1]) was obtained from the Protein Data Bank (PDB, www.rcsb.org). Chain A, corresponding to the Maltose-binding periplasmic protein and trichosatin A were deleted. All heteroatom records were removed, except for the metal ions (one zinc atom and two potassium atoms). Phenylhydroxamates with sterically demanding cap groups are known to bind via a Zn^{2+}-bound water molecule [1]. Considering this, a water molecule was introduced. Coordinates were identified from the aligned CD2-Domain of zHDAC6 (PDB: 6CGP [2]). The structure was optimized to the closest local energy minimum using RosettaRelax with coordinate constraints on the backbone and metal ion restraints [3]. Ligand input files (α)-**7c** and (β)-**7c** were created with ChemDraw. An initial 3D conformer with hydrogen atoms was constructed in Chem3D and energetically minimized using the MM2 force field, followed by the production of an ensemble of 1000 low-energy conformers with BCL:ConformerGenerator [4]. One conformer was placed in the binding pocket of HDAC6. A constraint file was constructed using the known distance of the zinc binding of trichostatin A [1]. Ligand docking was performed with RosettaLigand for an initial 5000 models. Those models were clustered according to their similarity in their binding mode. The depicted models are the best scoring models from the highest populated clusters [5–7].

Rosetta version 3.12 was used. The following commands were executed throughout the modeling process:

- Relax input starting structures:

```
rosetta/source/bin/relax.linuxgccrelease -s 5EDU_prepared.pdb rosetta/source/database/
-constrain_relax_to_start_coords -in:auto_setup_metals -nstruct 25 -out:prefix relaxed_
-ignore_unrecognized_res -overwrite -ignore_waters False
```

- Options for RosettaLigand:

```
-in:file:extra_res_fa ligand.params
-packing
        -ex1
        -ex2
        -no_optH false
        -flip_HNQ true
        -ignore_ligand_chi true


-parser
        -protocol dock.xml
```

```
-mistakes
    -restore_pre_talaris_2013_behavior true

-constraints:cst_file constraint_5EDU_hydroxamicacid.cst

-out:path:all ../output/
```

- RosettaScripts protocol for executing RosettaLigand:

```
<ROSETTASCRIPTS>
        <SCOREFXNS>
                <ScoreFunction name="ligand_soft_rep" weights="ligand_soft_rep">
                </ScoreFunction>
                <ScoreFunction name="hard_rep" weights="ligand">
                </ScoreFunction>
        </SCOREFXNS>
        <LIGAND_AREAS>
                <LigandArea name="inhibitor_dock_sc" chain="X" cutoff="6.0"
                add_nbr_radius="true"all_atom_mode="false"/>
                <LigandArea name="inhibitor_final_sc" chain="X" cutoff="6.0"
                add_nbr_radius="true" all_atom_mode="false"/>
                <LigandArea name="inhibitor_final_bb" chain="X" cutoff="7.0"
                add_nbr_radius="false" all_atom_mode="true"  alpha_restraints="0.3"/>
        </LIGAND_AREAS>
        <INTERFACE_BUILDERS>
                <InterfaceBuilder name="side_chain_for_docking"
                ligand_areas="inhibitor_dock_sc"/>
                <InterfaceBuilder name="side_chain_for_final" ligand_areas="inhibitor_final_sc"/>
                <InterfaceBuilder name="backbone"
                ligand_areas="inhibitor_final_bb"extension_window="3"/>
        </INTERFACE_BUILDERS>
        <MOVEMAP_BUILDERS>
                <MoveMapBuilder name="docking"
                sc_interface="side_chain_for_docking"minimize_water="false"/>
                <MoveMapBuilder name="final"
                sc_interface="side_chain_for_final"bb_interface="backbone"minimize_water="false"\
                >
        </MOVEMAP_BUILDERS>
        <SCORINGGRIDS ligand_chain="X" width="15">
```

```
<ClassicGrid grid_name="classic" weight="1.0"/>

</SCORINGGRIDS>

<MOVERS>

<StartFrom name="startFrom" chain="X">

<Coordinates x="17.12" y="-44.26" z="102.7"/>

</StartFrom>

<ConstraintSetMover name="constraint" add_constraints="true"cst_file="./
constraint_5EDU_hydroxamicacid.cst"/>
<Transform name="transform" chain="X" box_size="7.0" move_distance="0.2"
angle="20" cycles="500" repeats="1" temperature="5"/>

<HighResDocker name="high_res_docker" cycles="6"
repack_every_Nth="3"scorefxn="ligand_soft_rep"movemap_builder="docking"/>

<FinalMinimizer name="final" scorefxn="hard_rep" movemap_builder="final"/>

<ClearConstraintsMover name="clearconstraints"/>

<InterfaceScoreCalculator name="add_scores" chains="X" scorefxn="hard_rep"/>

</MOVERS>

<PROTOCOLS>

<Add mover_name="startFrom"/>
<Add mover_name="constraint"/>
<Add mover_name="transform"/>
<Add mover_name="high_res_docker"/>
<Add mover_name="final"/>
<Add mover_name="clearconstraints"/>
<Add mover_name="add_scores"/>

</PROTOCOLS>

</ROSETTASCRIPTS>
```

- Executing RosettaLigand:

```
cat ./listof_pdbs_5EDU | parallel -j 10 bash run_dock_parallel.sh
```

Ligand RMSD vs Interface Score plots of

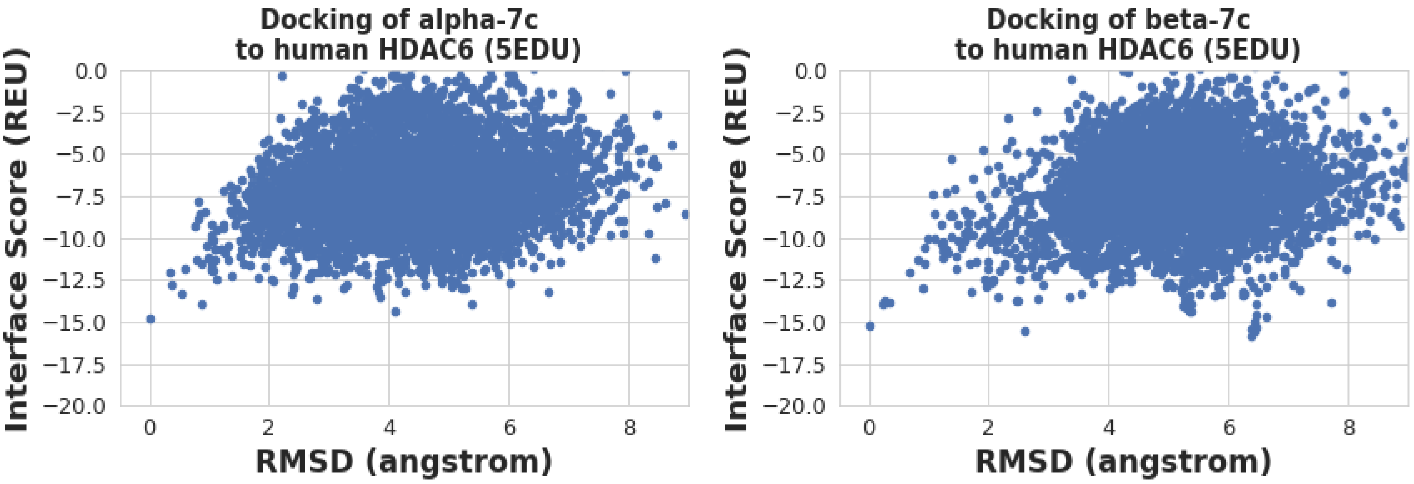

Ligand RMSD vs Interface Score plots for (α)-**7c** and (β)-**7c**. It was plotted against the models with the best scoring from the highest populated clusters.

3. ^{1}H and ^{13}C NMR spectra

^{1}H-NMR (DMSO-*d*$_6$, 400.2 MHz) of **6a**

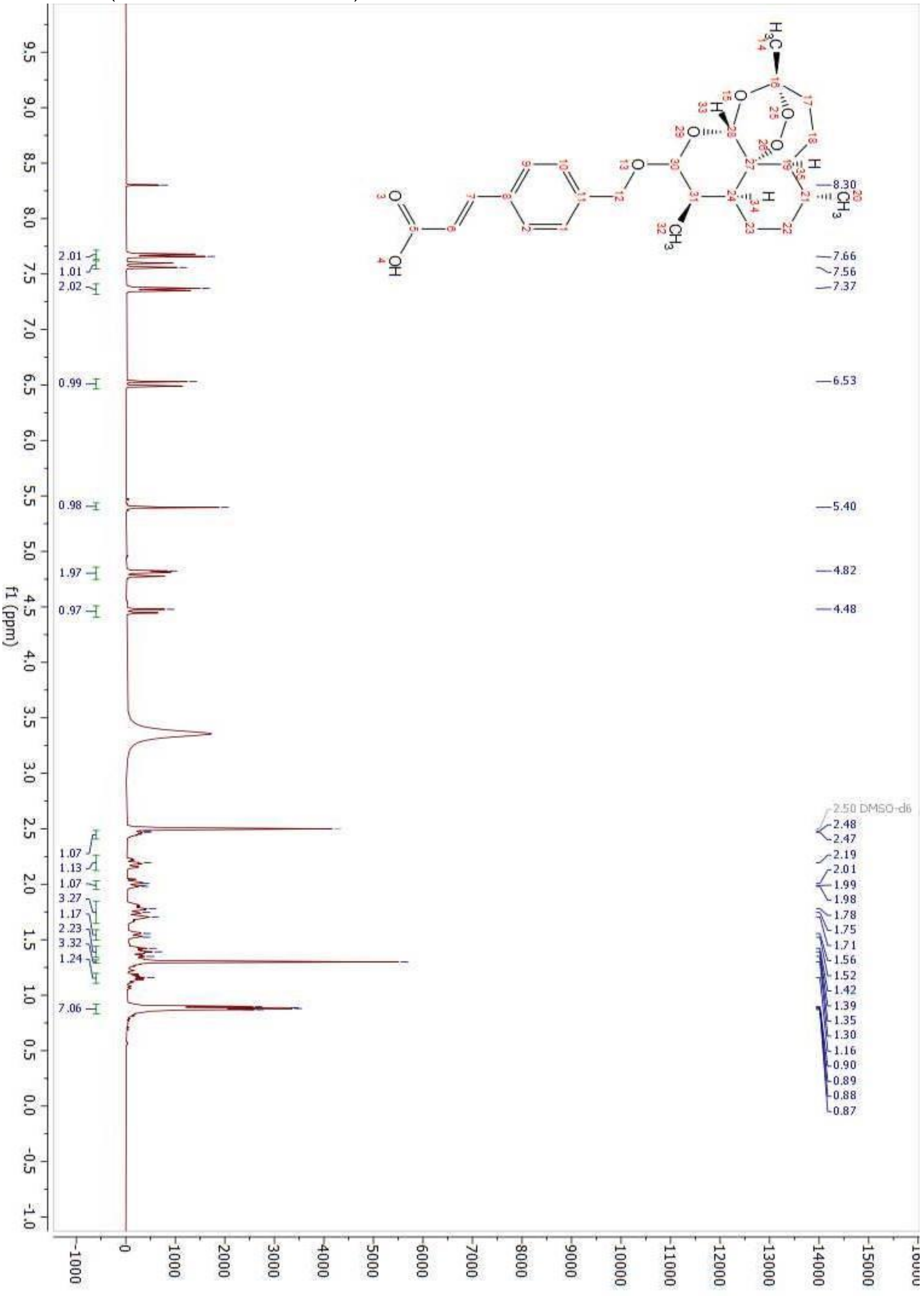

^{13}C-NMR (DMSO-*d*$_6$, 100.6 MHz) of **6a**

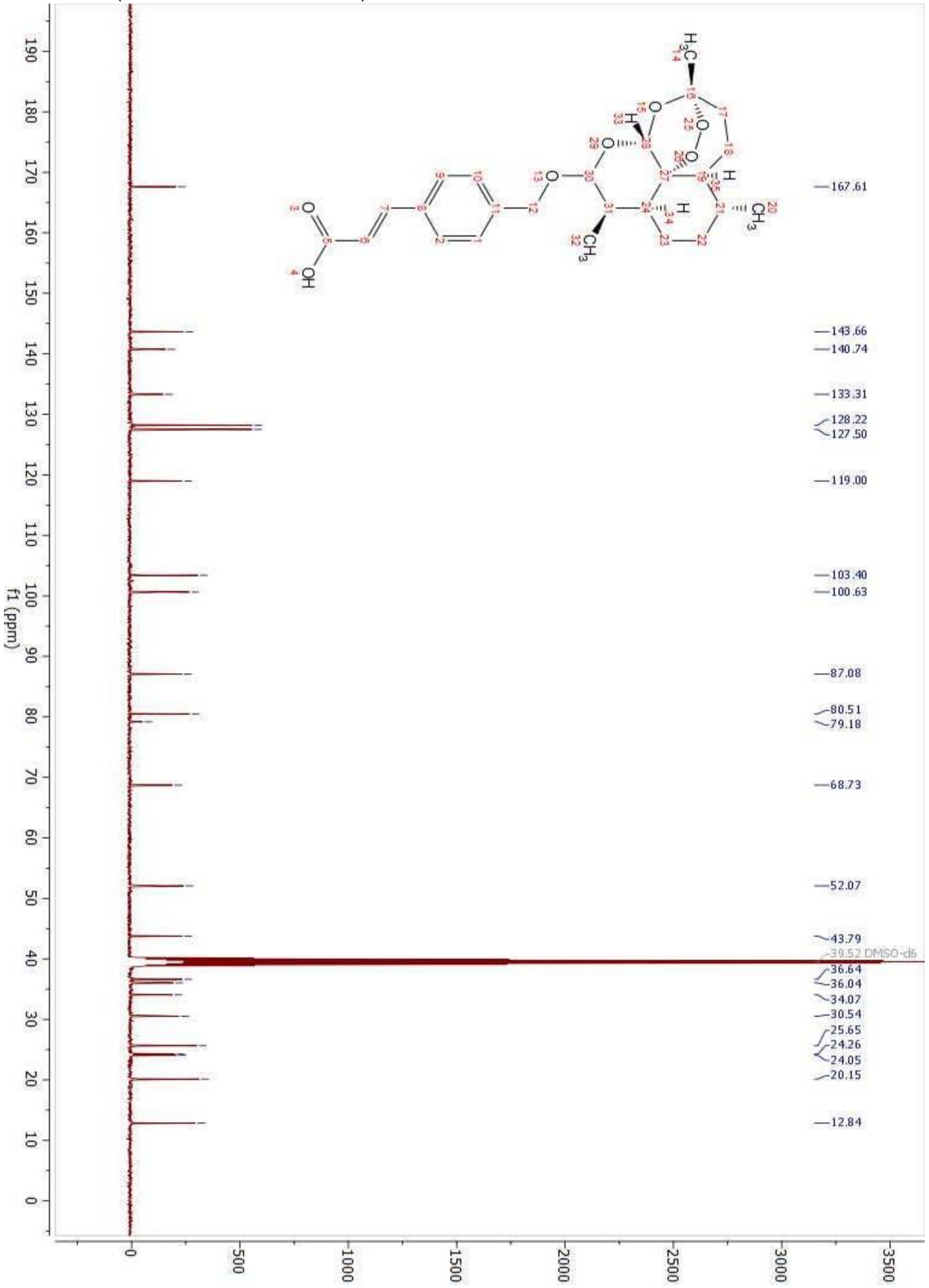

^{1}H-NMR (DMSO-d_6, 400.2 MHz) of **6b**

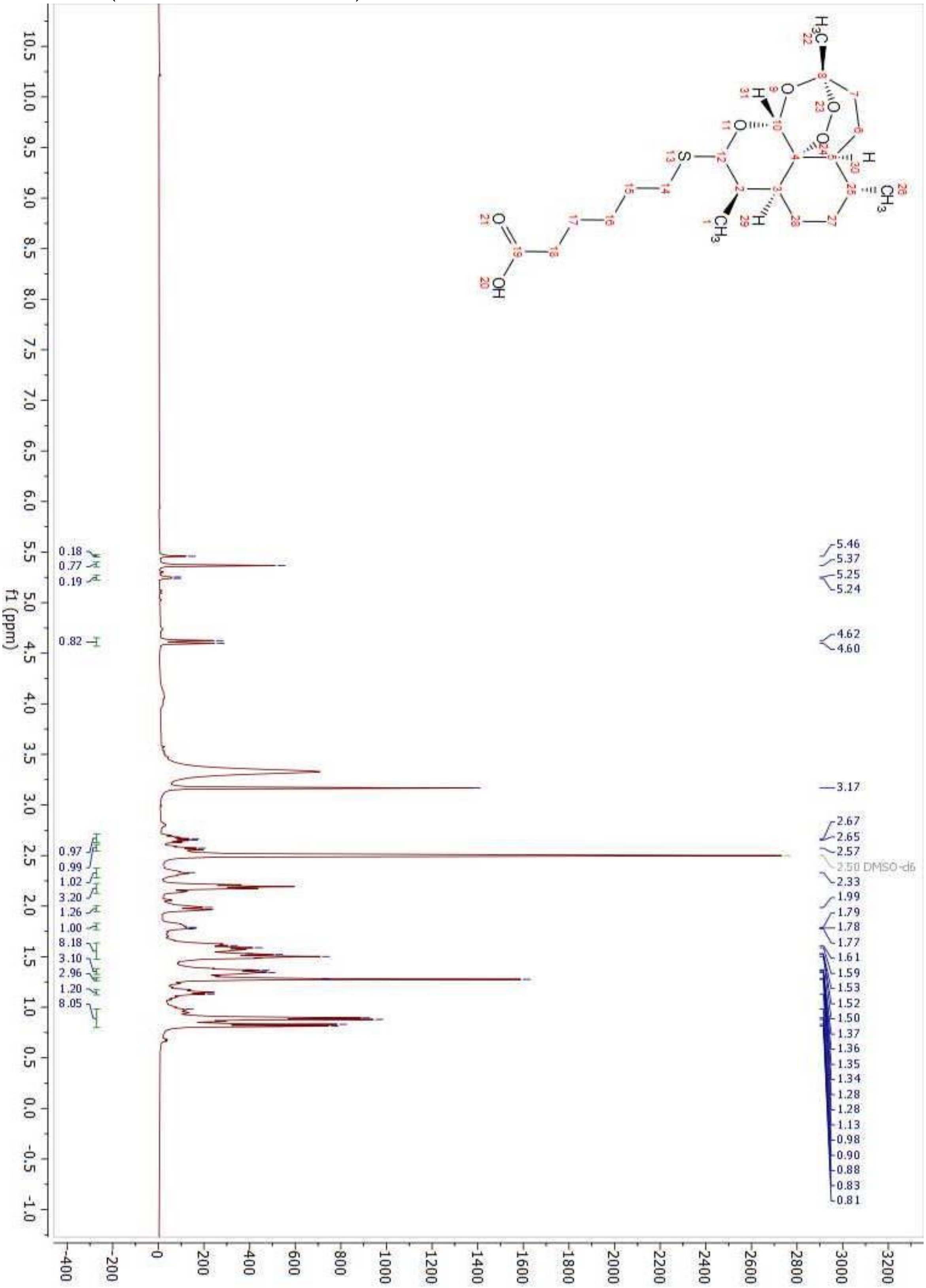

^{13}C-NMR (DMSO-*d*6, 100.6 MHz) of **6b**

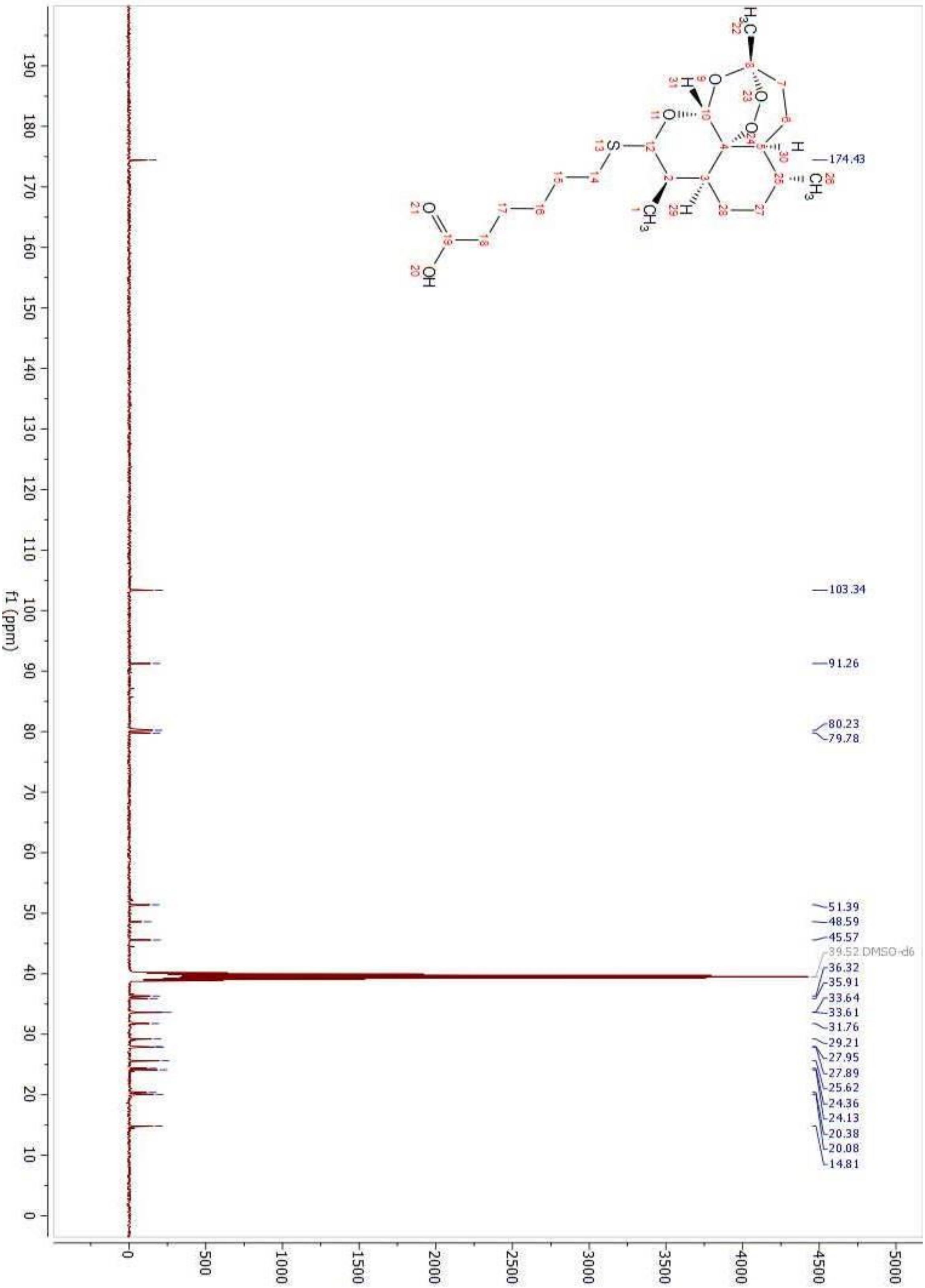

^{1}H-NMR (DMSO-d_6, 400.2 MHz) of **6c**

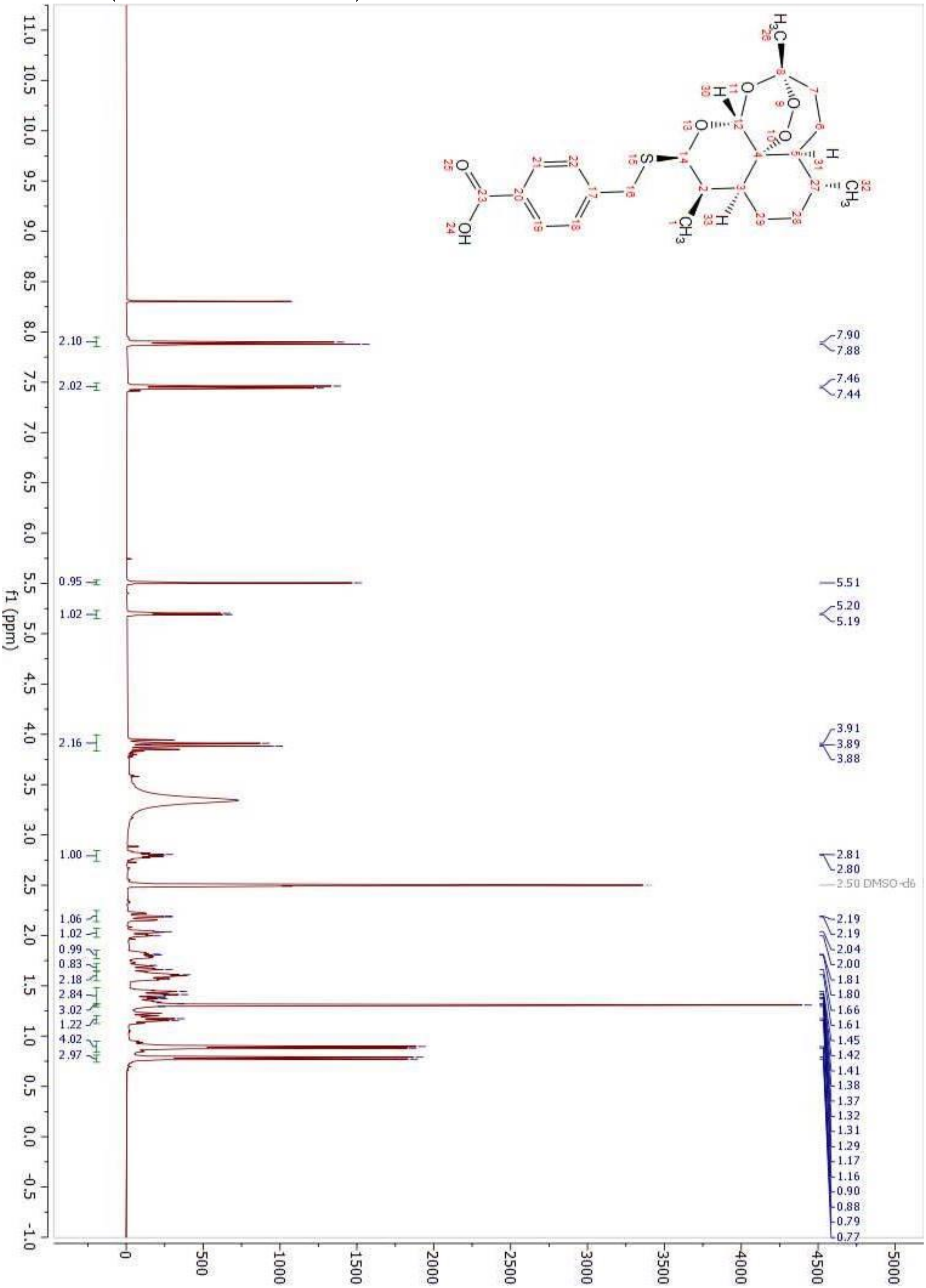

^{13}C-NMR (DMSO-d_6, 100.6 MHz) of **6c**

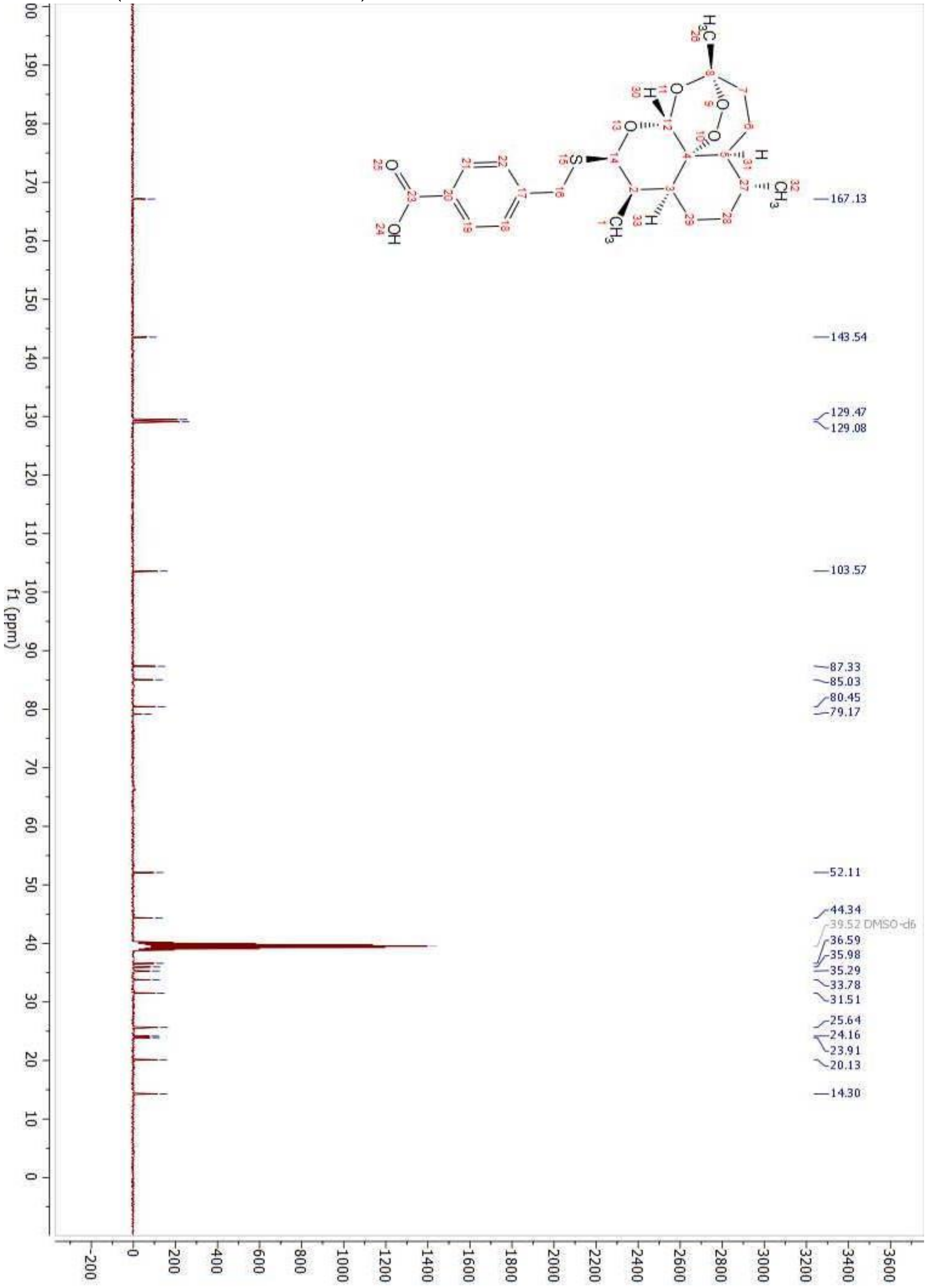

^{1}H-NMR (DMSO-d_6, 300.1 MHz) of **6d**

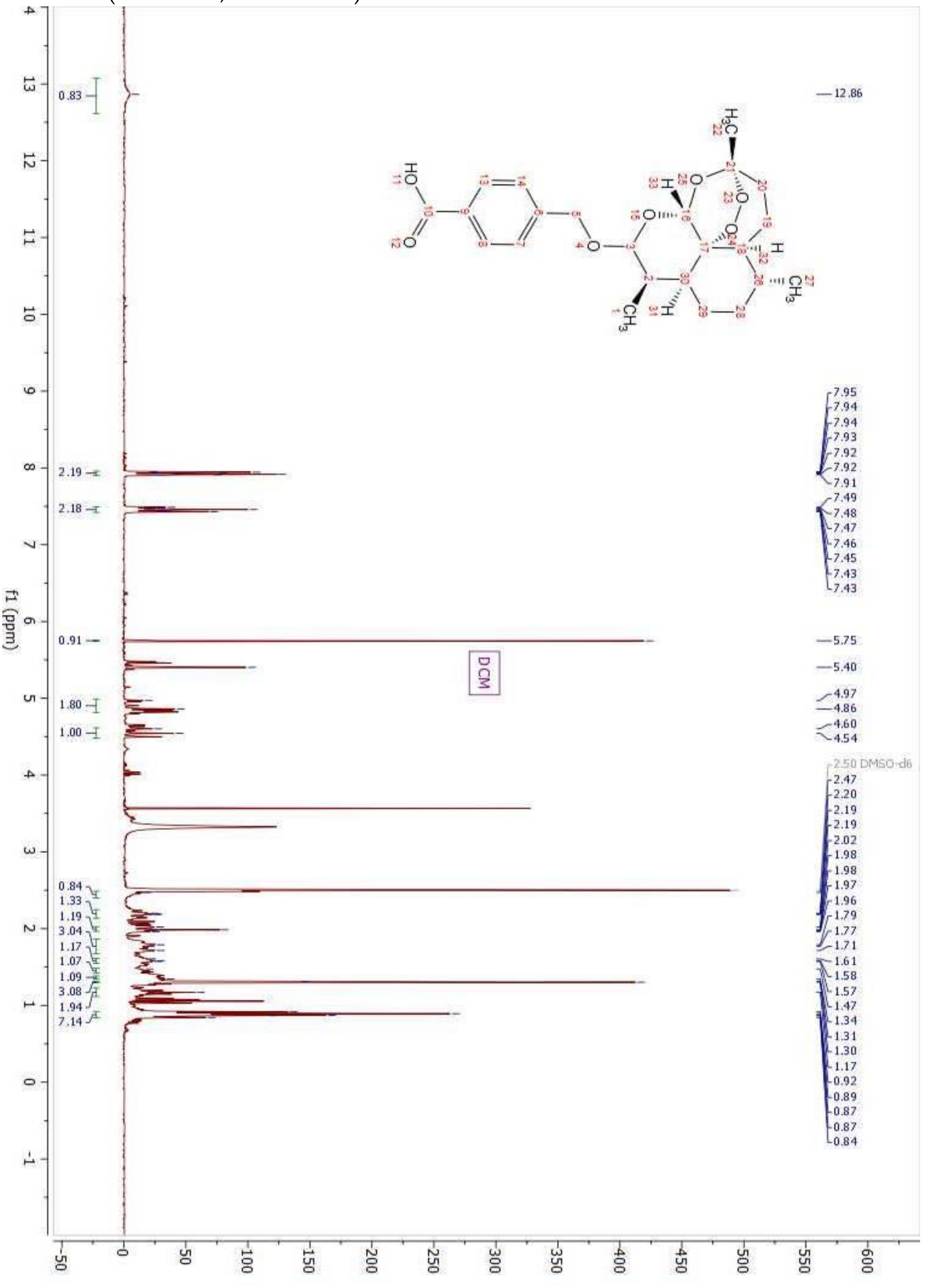

^{13}C-NMR (DMSO-*d*$_6$, 75.5 MHz) of **6d**

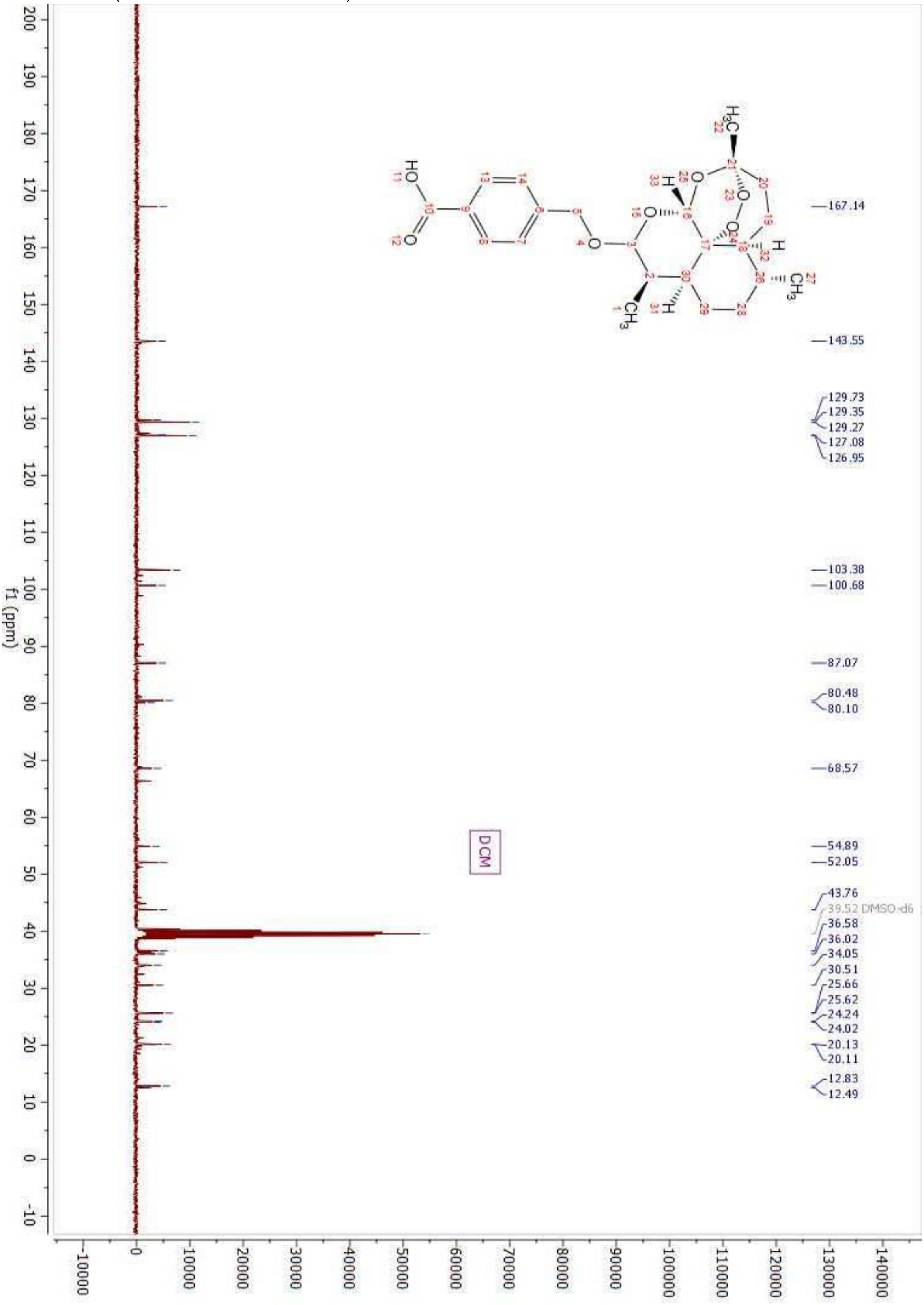

^{1}H-NMR ($CDCl_3$, 300.1 MHz) of **7a**

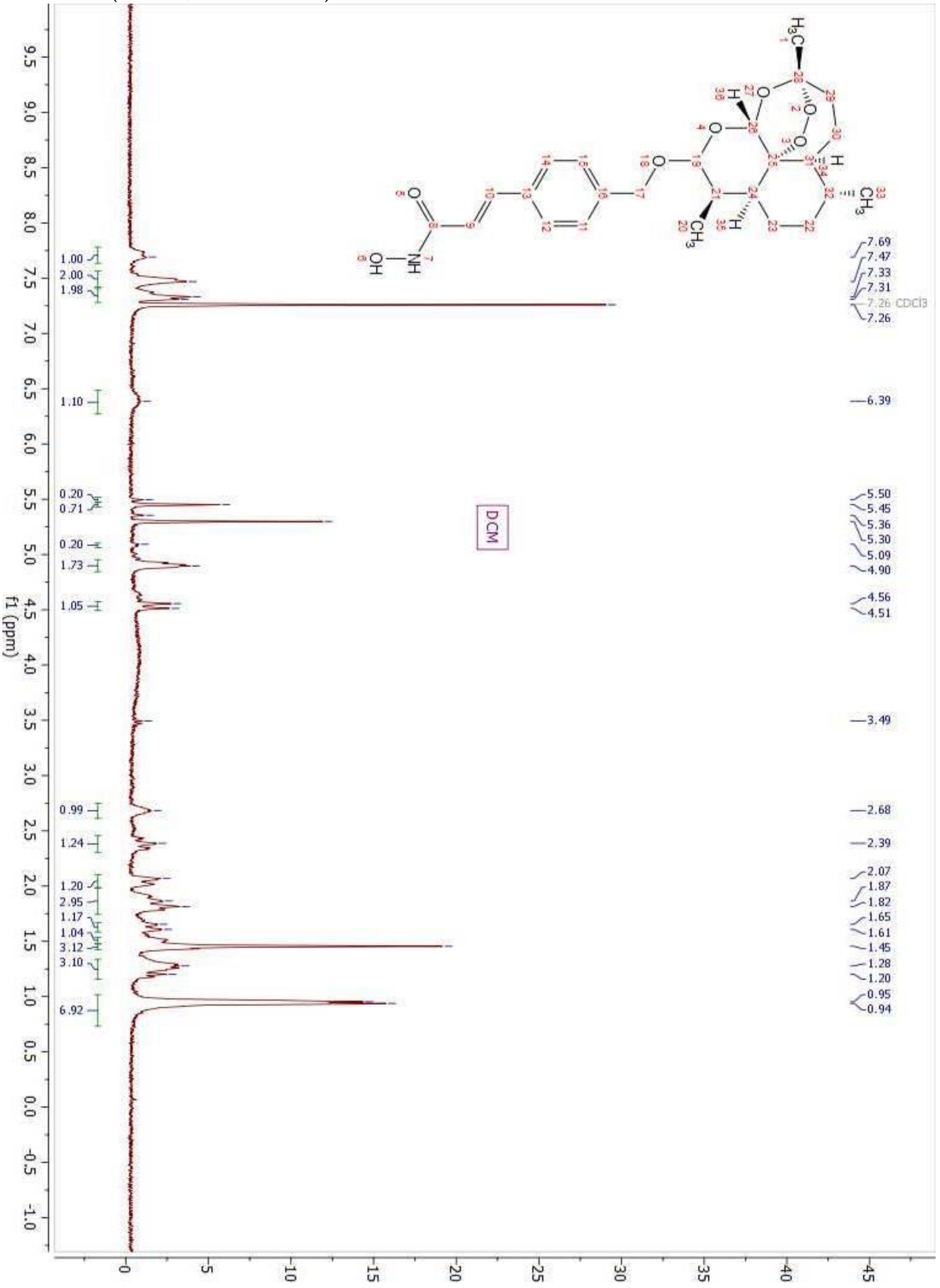

^{13}C-NMR (DMSO-d_6, 100.6 MHz) of **7a**

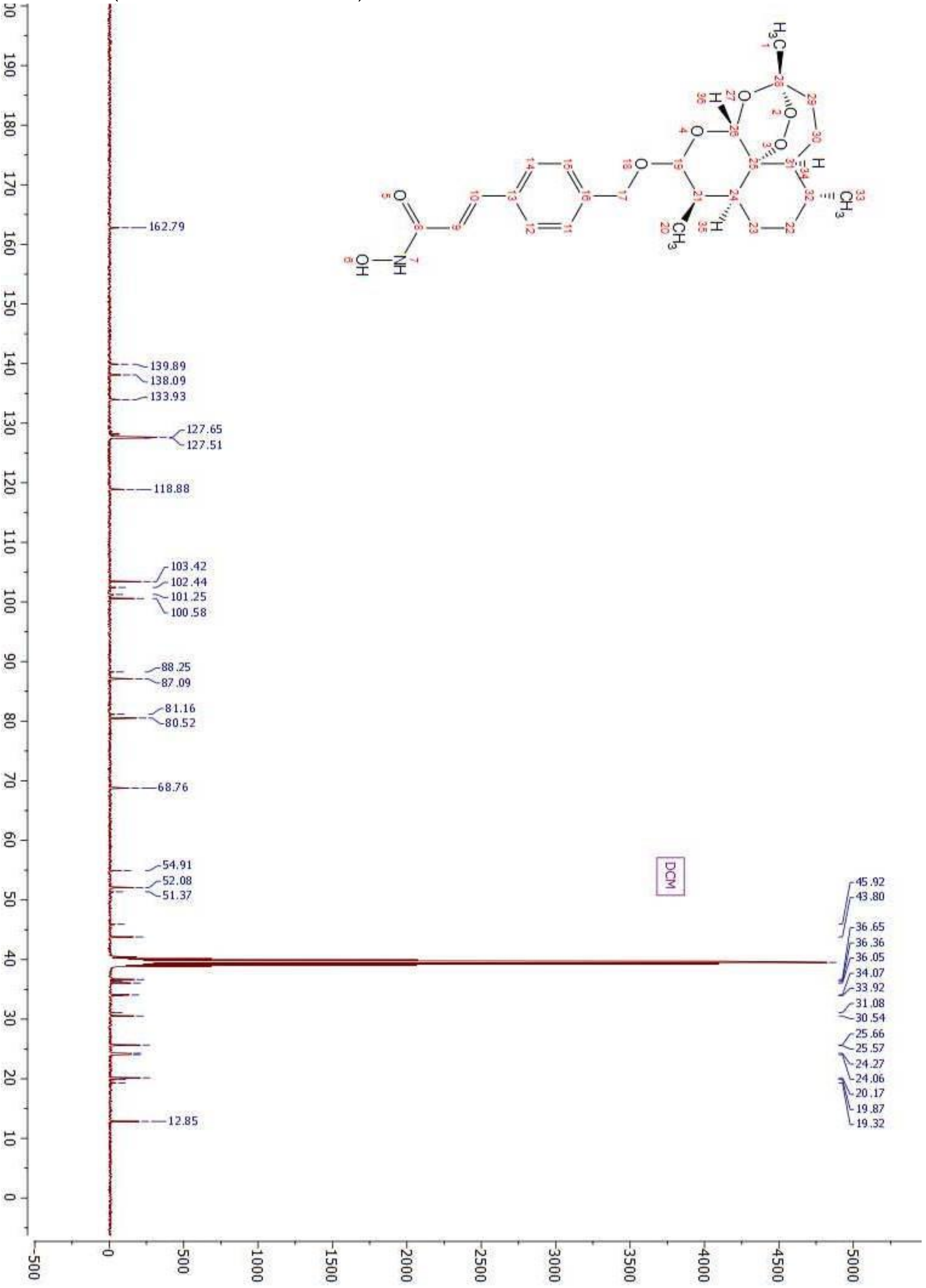

^{1}H-NMR ($CDCl_3$, 400.2 MHz) of **7b**

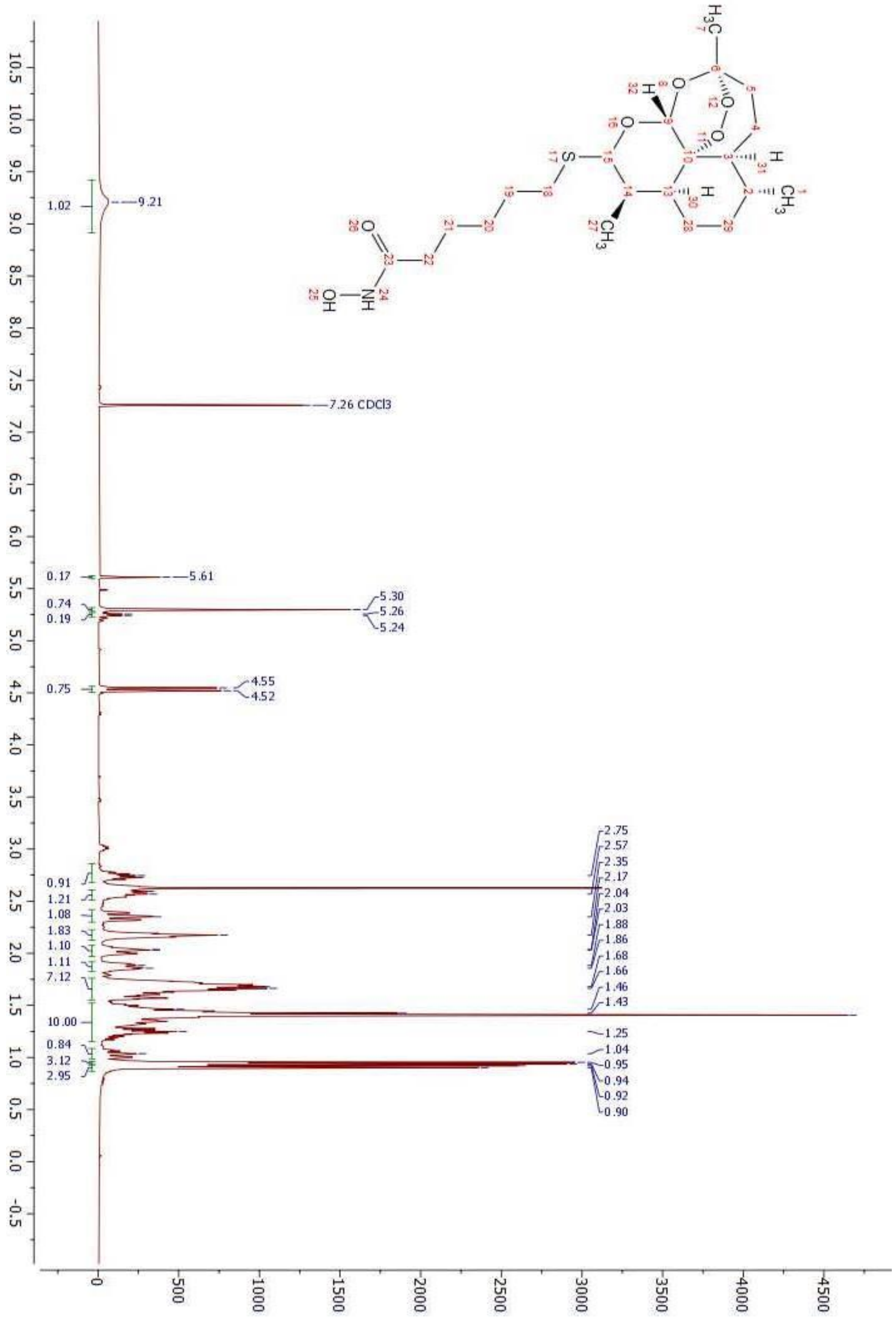

^{13}C-NMR ($CDCl_3$, 100.6 MHz) of **7b**

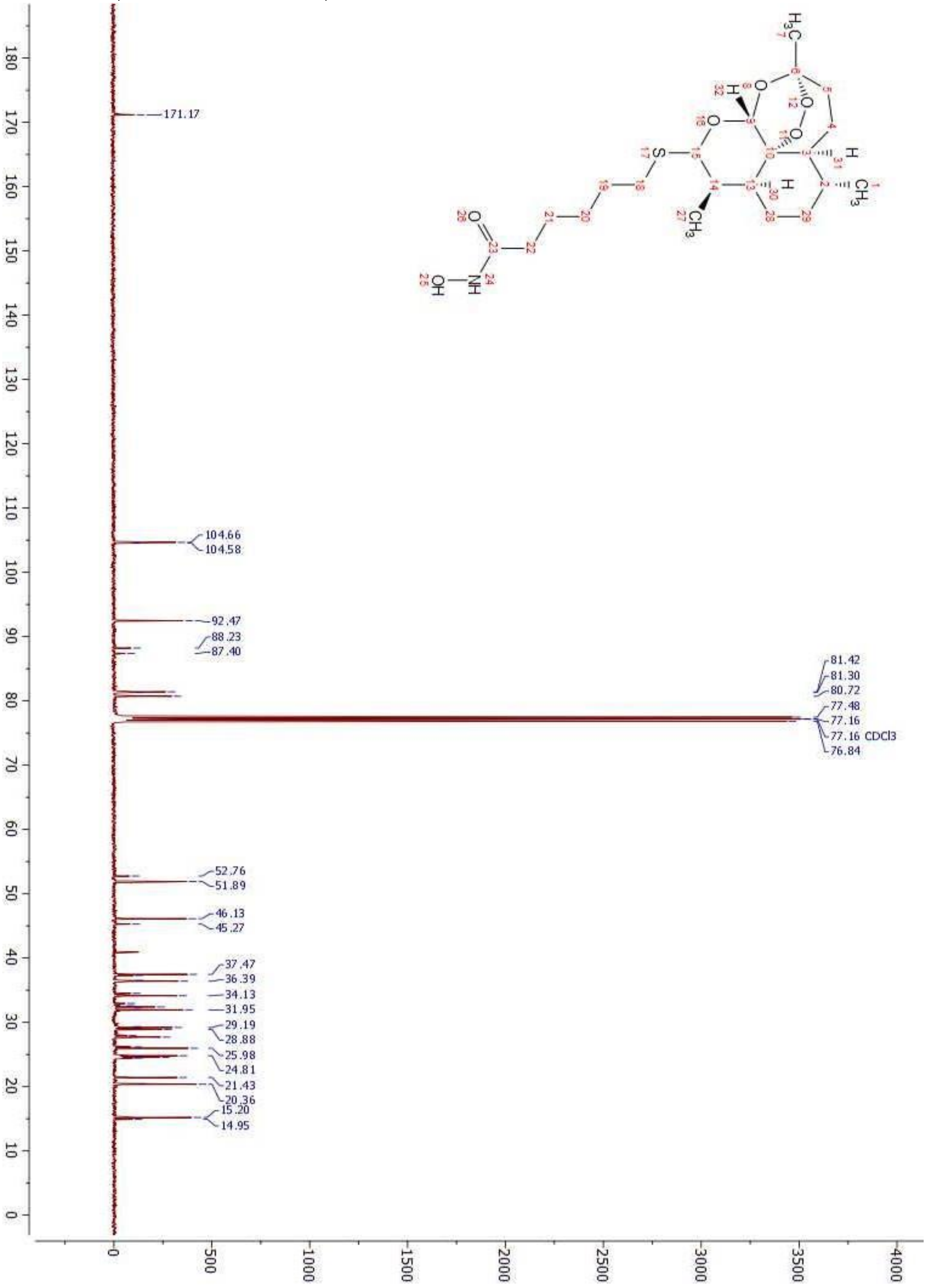

^{1}H-NMR (DMSO-d_6, 400.2 MHz) of (α)-**7c**

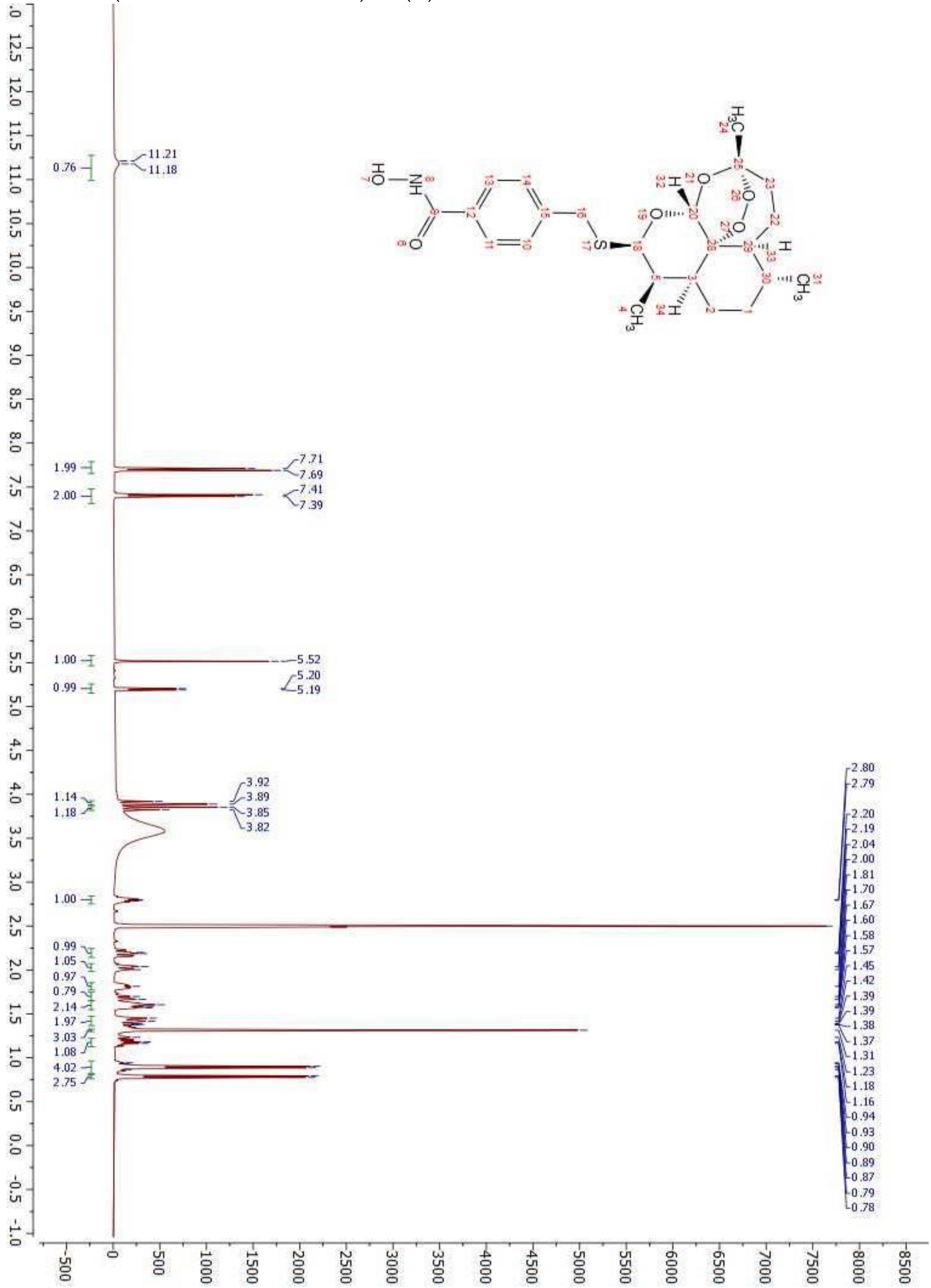

^{13}C-NMR ($CDCl_3$, 100.6 MHz) of (α)-**7c**

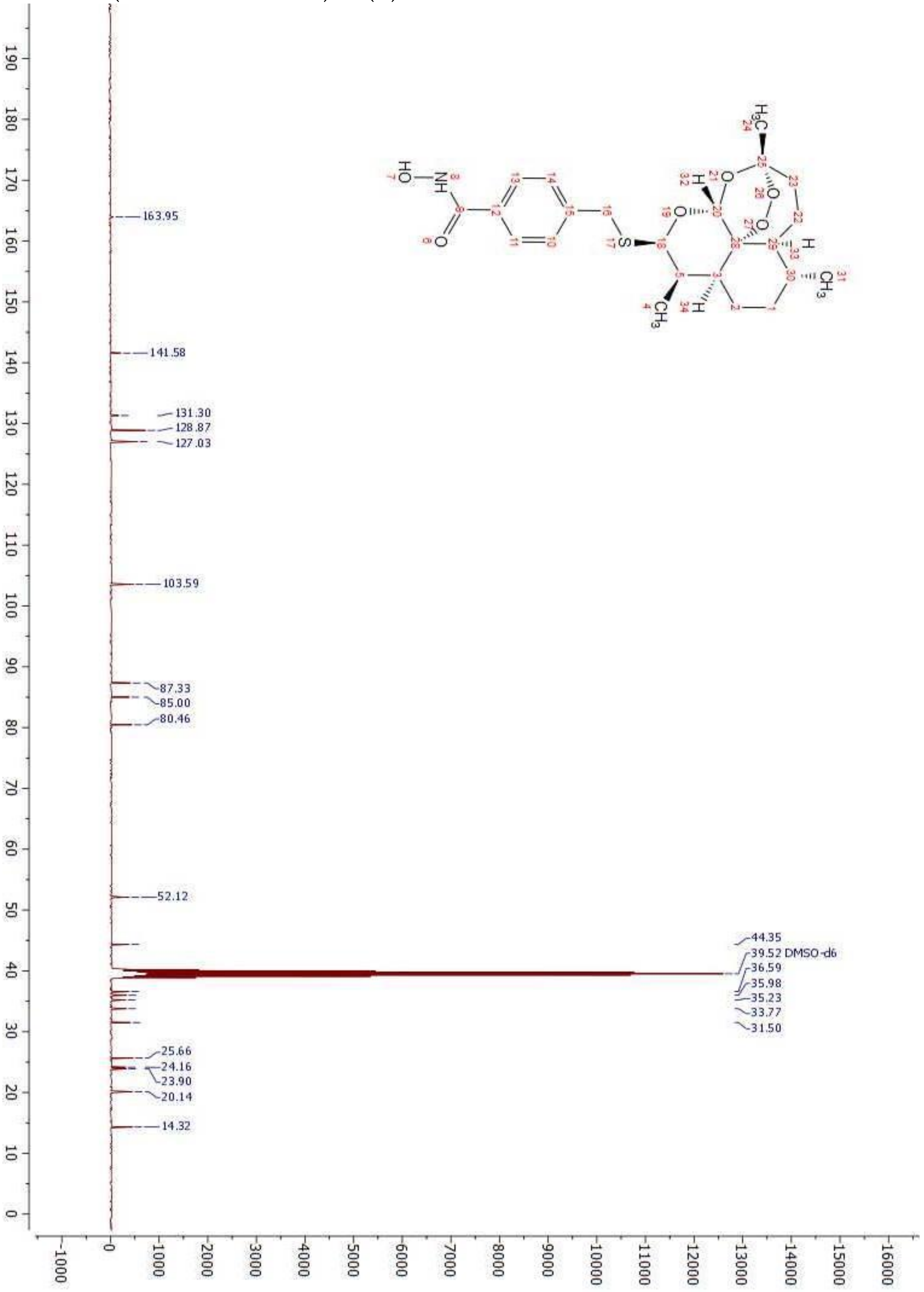

^{1}H-NMR (DMSO-d_6, 400.2 MHz) of (β)-**7c**

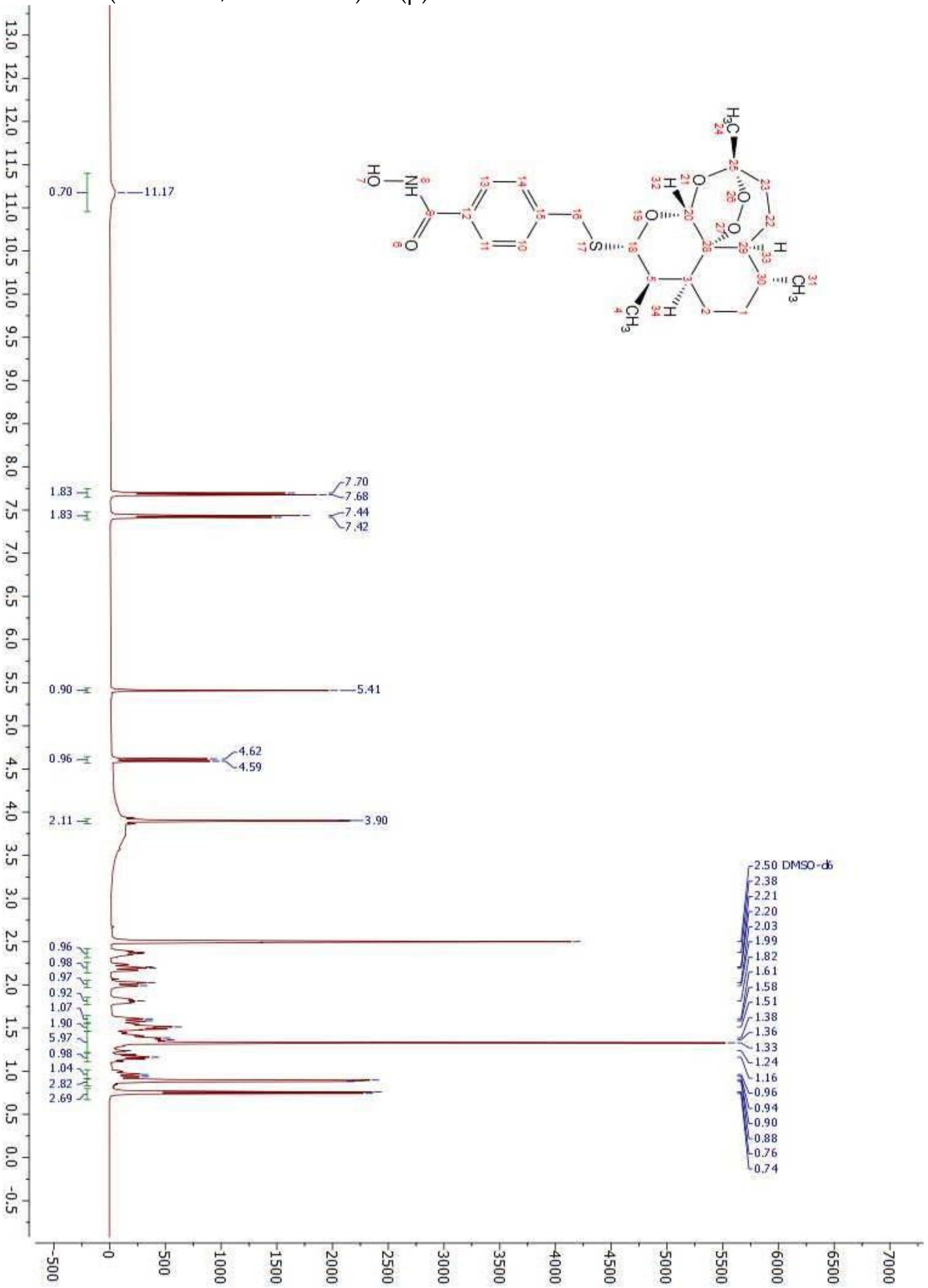

^{13}C-NMR ($CDCl_3$, 100.6 MHz) of (β)-**7c**

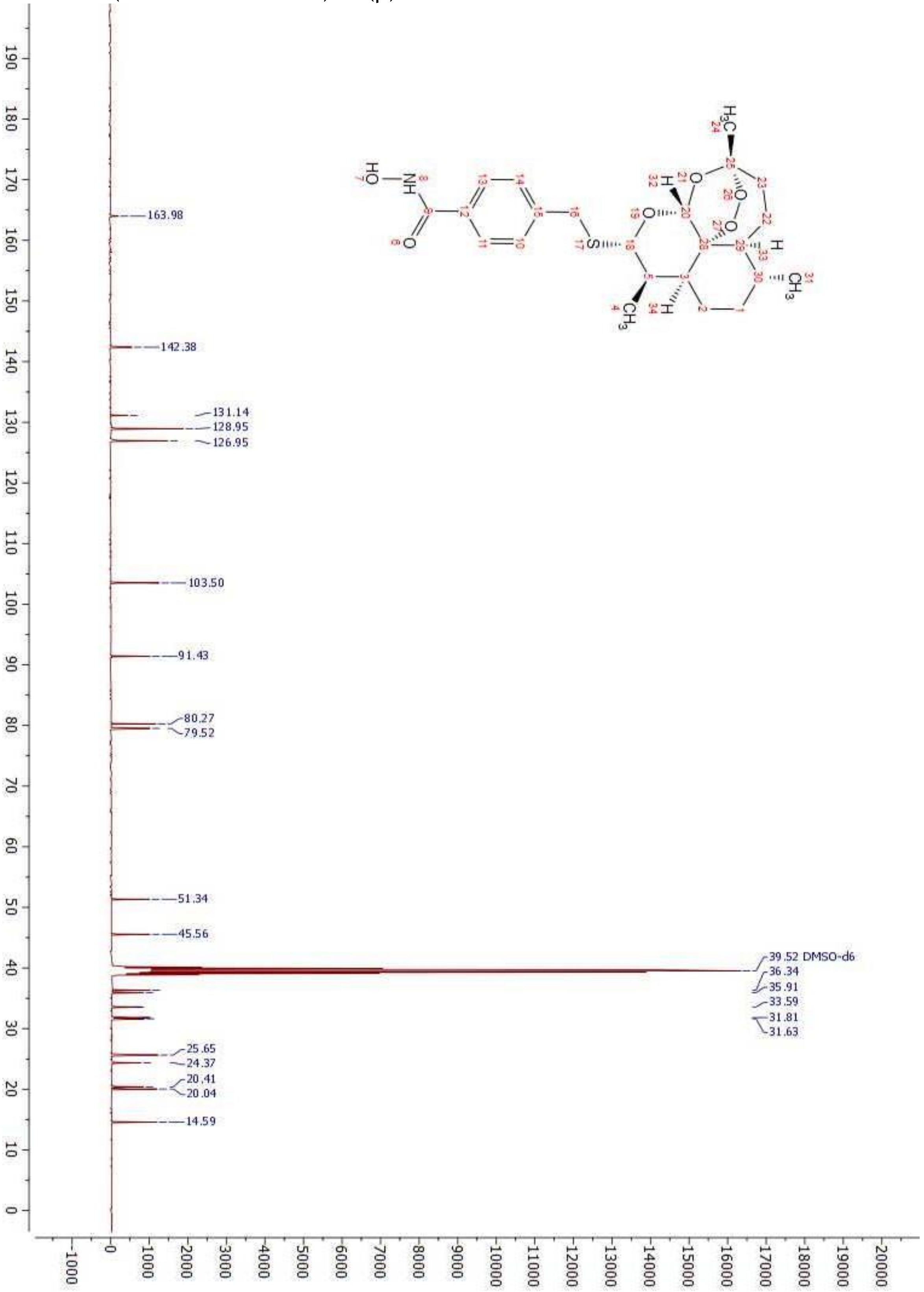

^{1}H-NMR (DMSO-d_6, 300.8 MHz) of **7d**

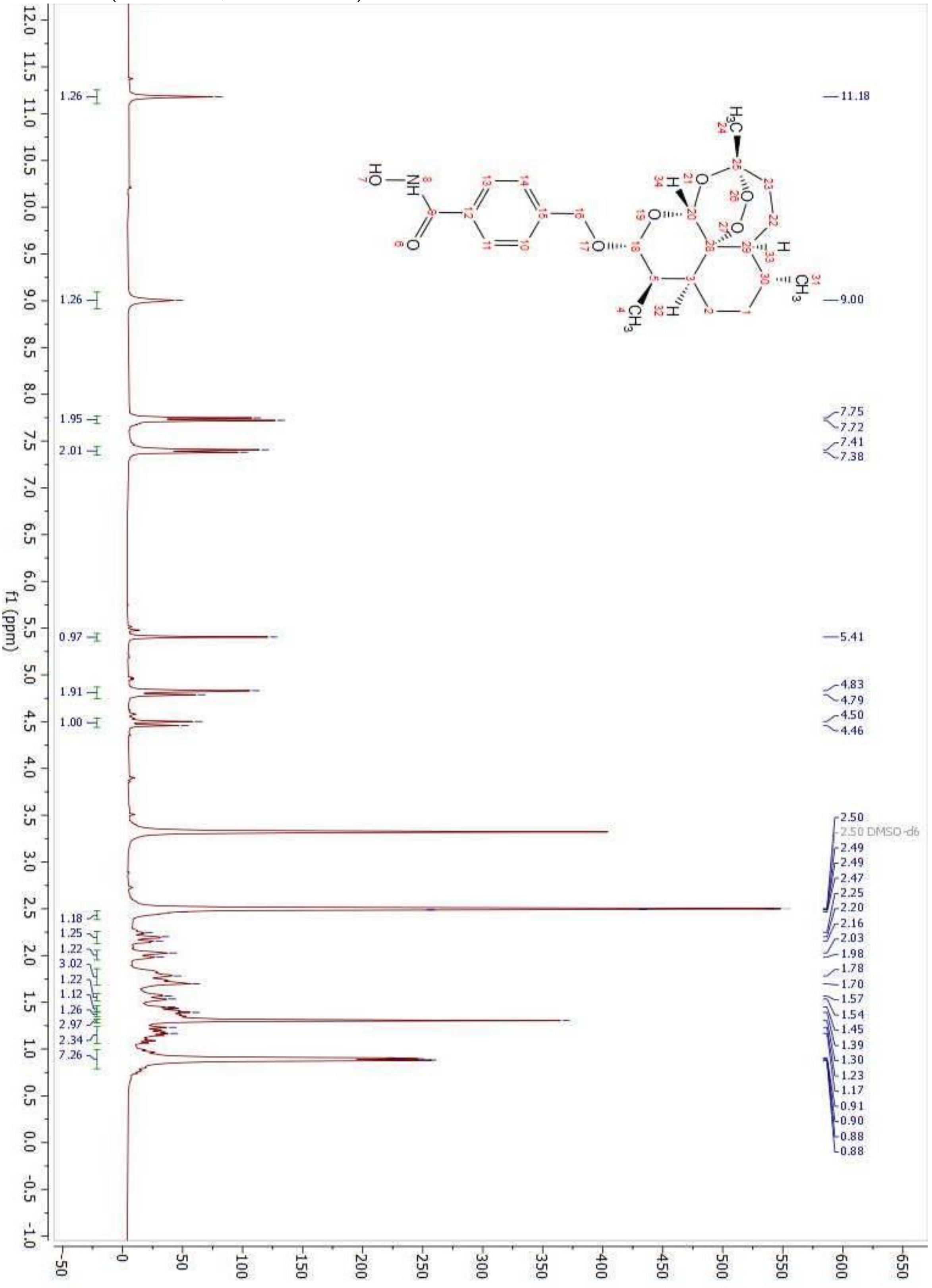

^{13}C-NMR (DMSO-d_6, 75.5 MHz) of **7d**

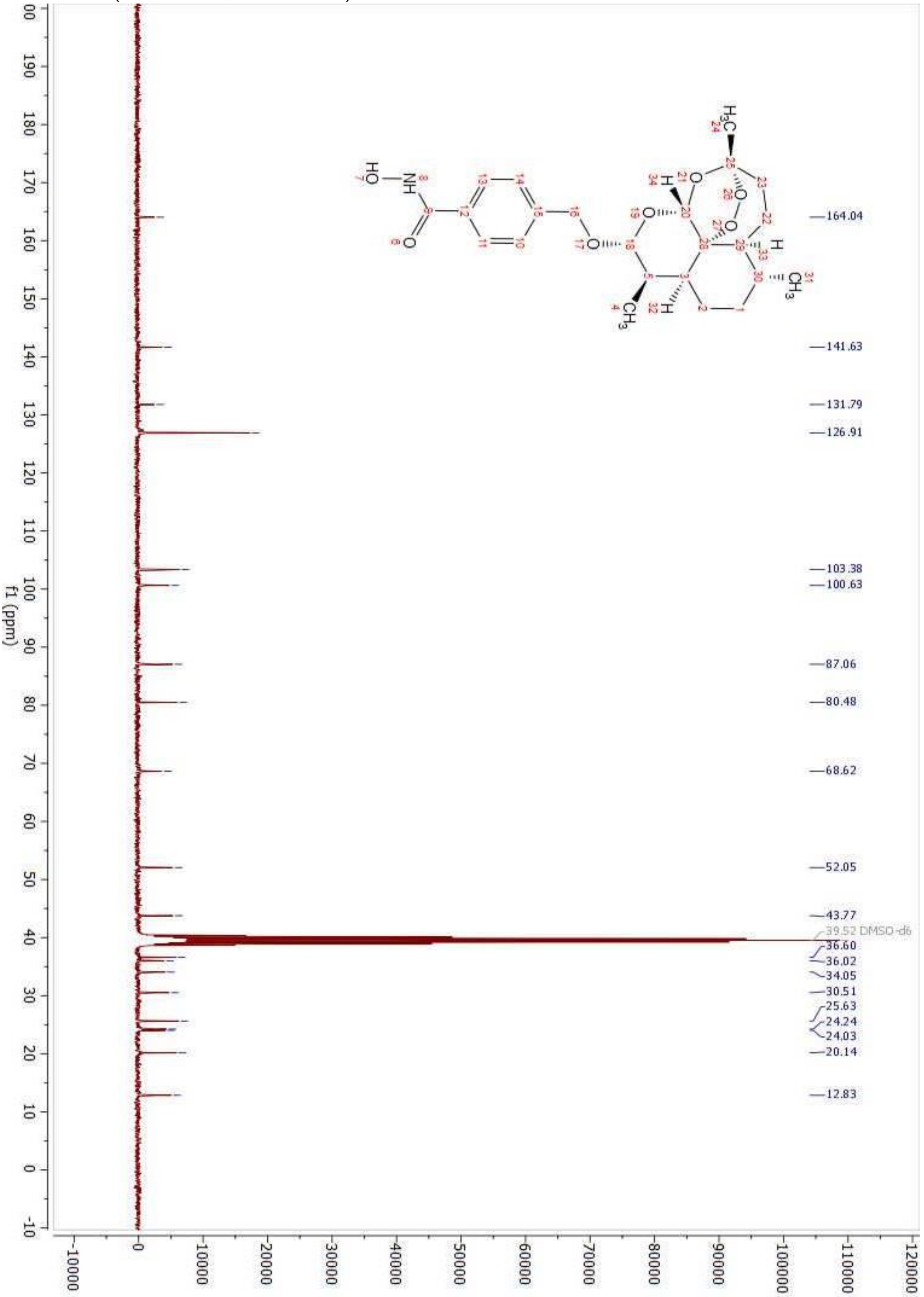

^{1}H-NMR (DMSO-d_6, 300.1 MHz) of (α)-**7e**

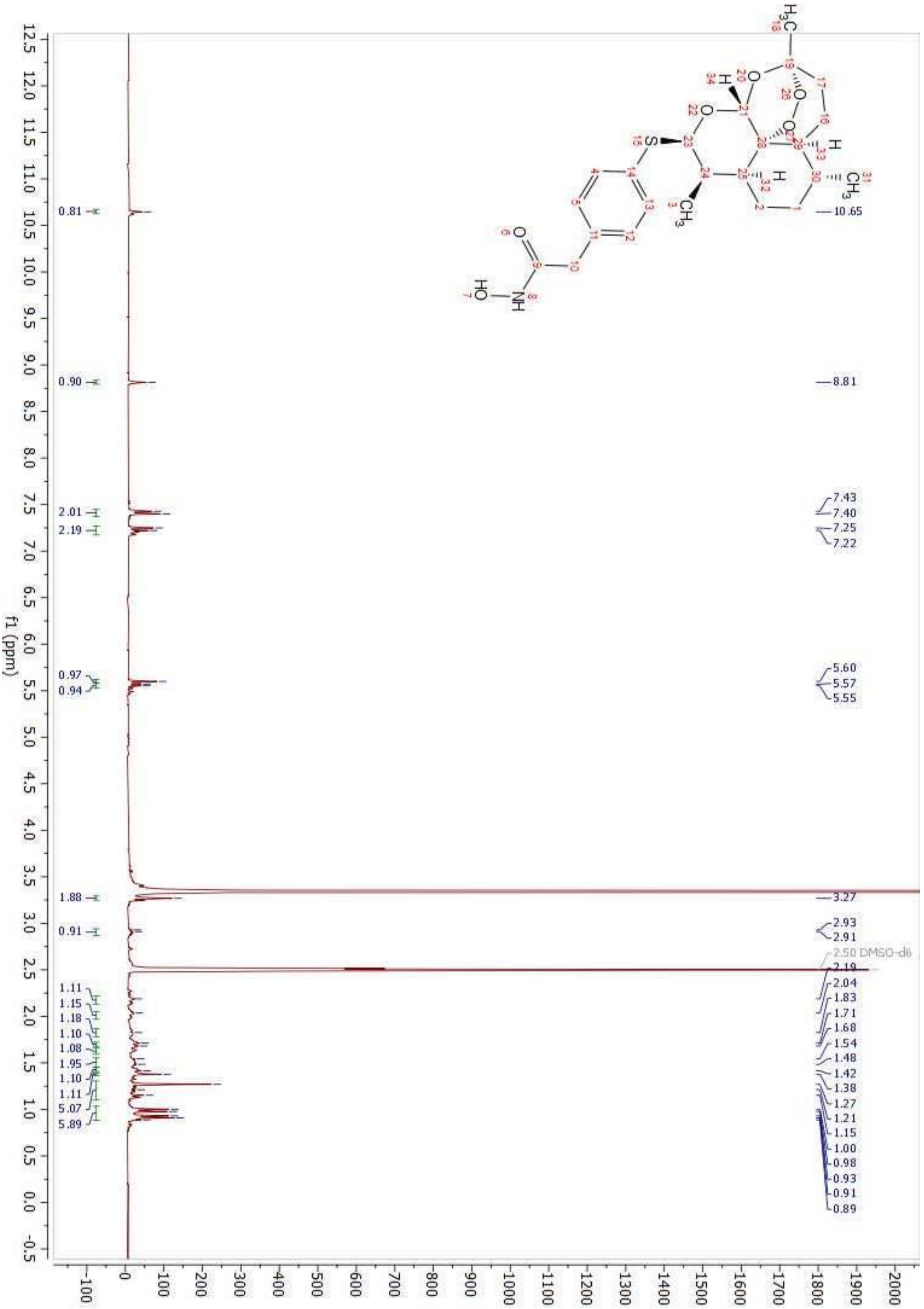

^{1}H-NMR (DMSO-d_6, 400.0 MHz) of (β)-**7e**

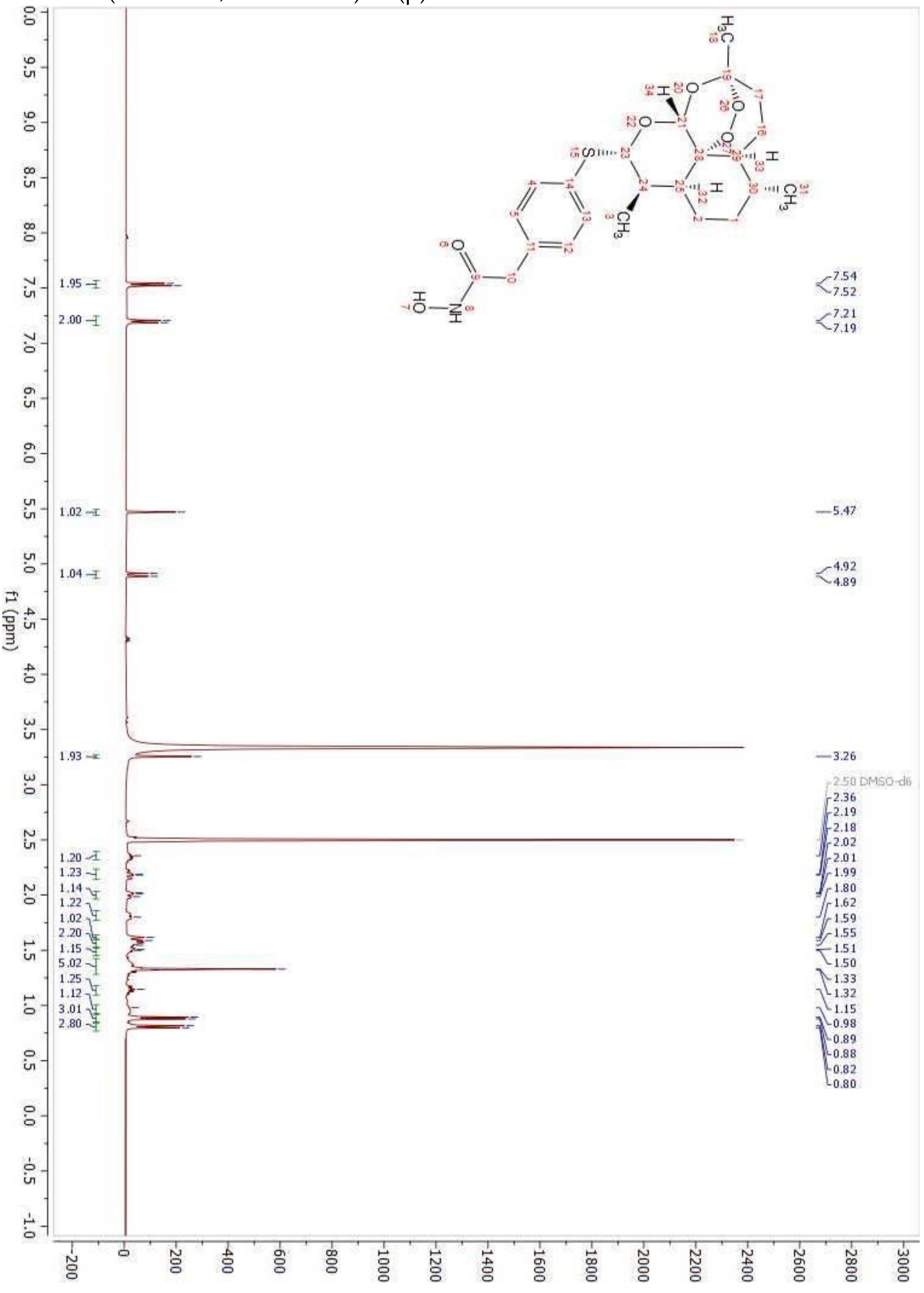

^{13}C-NMR (DMSO-d_6, 100.6 MHz) of (β)-**7e**

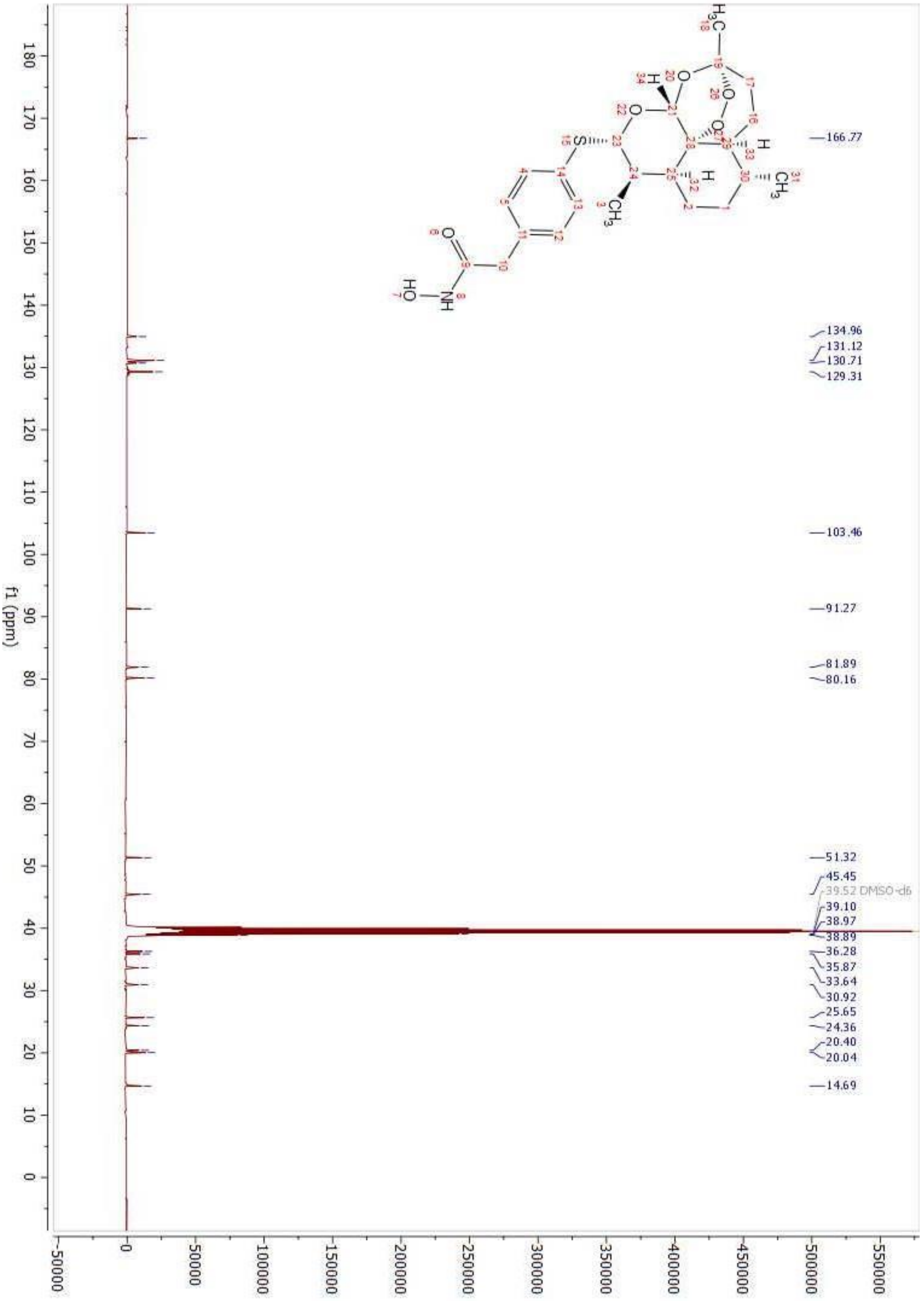

^{1}H-NMR ($CDCl_3$, 400.2 MHz) of **8**

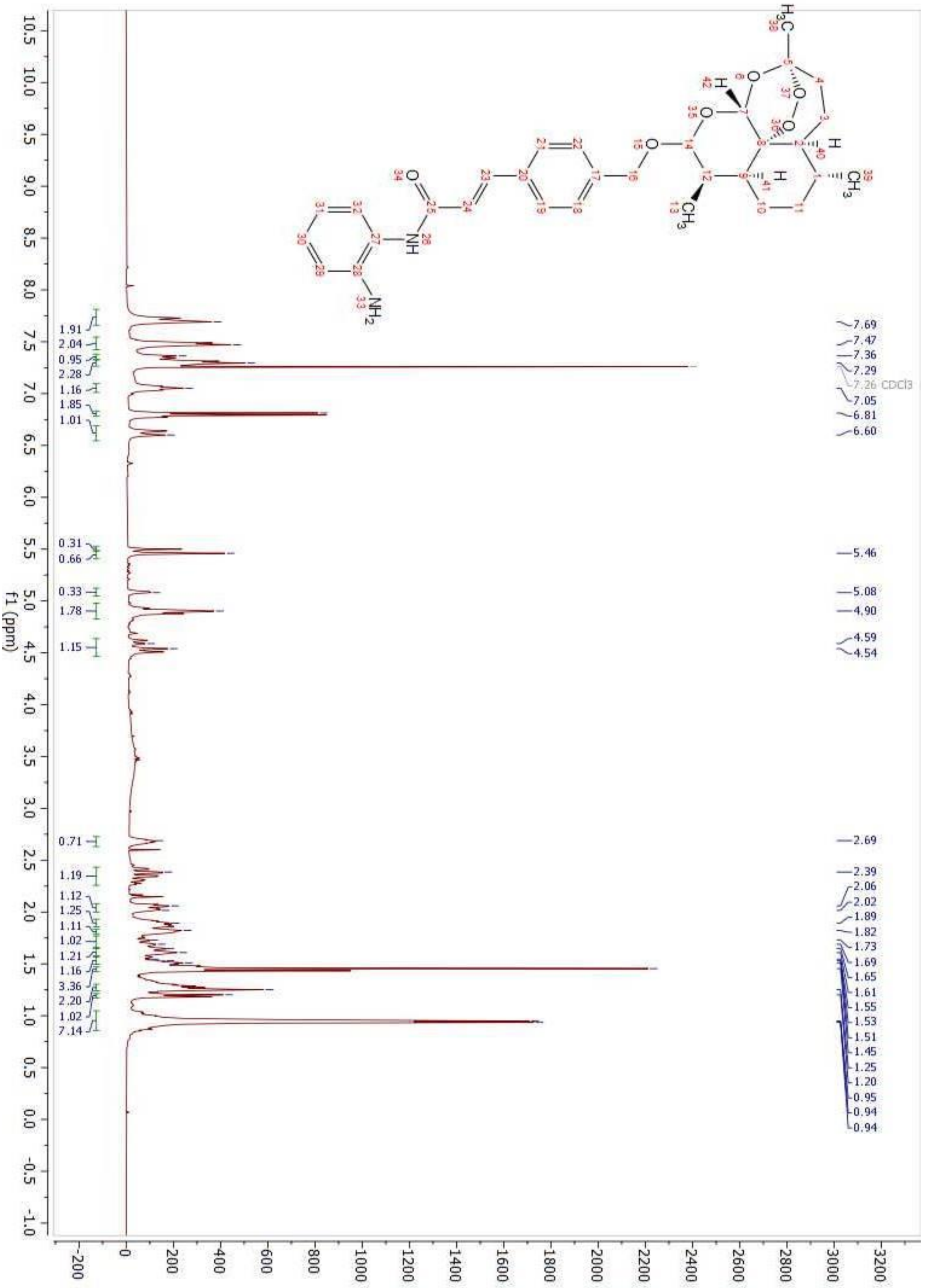

^{13}C-NMR ($CDCl_3$, 100.6 MHz) of **8**

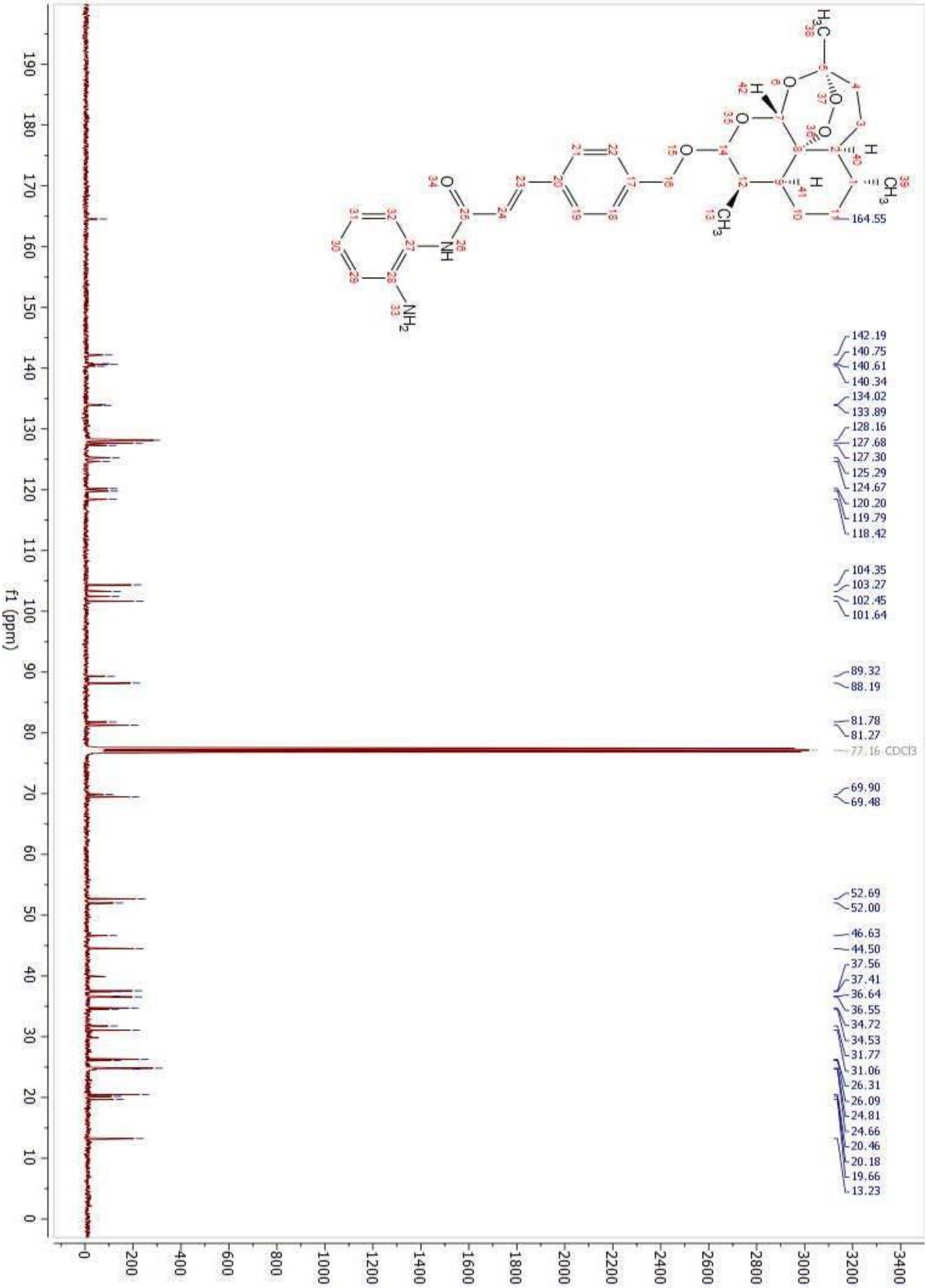

4. References

1. Hai, Y.; Christianson, D.W. Histone deacetylase 6 structure and molecular basis of catalysis and inhibition. *Nat. Chem. Biol.* **2016**, *12*, 741–747, doi:10.1038/nchembio.2134.
2. Mackwitz, M.K.W.; Hamacher, A.; Osko, J.D.; Held, J.; Schöler, A.; Christianson, D.W.; Kassack, M.U.; Hansen, F.K. Multicomponent Synthesis and Binding Mode of Imidazo1,2- apyridine-Capped Selective HDAC6 Inhibitors. *Org. Lett.* **2018**, *20*, 3255–3258, doi:10.1021/acs.orglett.8b01118.
3. Nivón, L.G.; Moretti, R.; Baker, D. A Pareto-optimal refinement method for protein design scaffolds. *PLoS One* **2013**, *8*, e59004, doi:10.1371/journal.pone.0059004.
4. Kothiwale, S.; Mendenhall, J.L.; Meiler, J. BCL:Conf: small molecule conformational sampling using a knowledge based rotamer library. *J. Cheminform.* **2015**, *7*, 47, doi:10.1186/s13321-015-0095-1.
5. Meiler, J.; Baker, D. ROSETTALIGAND: protein-small molecule docking with full side-chain flexibility. *Proteins* **2006**, *65*, 538–548, doi:10.1002/prot.21086.
6. Kaufmann, K.W.; Meiler, J. Using RosettaLigand for small molecule docking into comparative models. *PLoS One* **2012**, *7*, e50769, doi:10.1371/journal.pone.0050769.
7. DeLuca, S.; Khar, K.; Meiler, J. Fully Flexible Docking of Medium Sized Ligand Libraries with RosettaLigand. *PLoS One* **2015**, *10*, e0132508, doi:10.1371/journal.pone.0132508.
